# Phytochemicals From *Vicia faba* Beans as Ligands of the Aryl Hydrocarbon Receptor to Regulate Autoimmune Diseases

**DOI:** 10.3389/fnut.2022.790440

**Published:** 2022-03-04

**Authors:** Luis Fernando Méndez-López, Deisy Sosa de León, Manuel López-Cabanillas Lomelí, Blanca Edelia González-Martínez, Jesús Alberto Vázquez-Rodríguez

**Affiliations:** Laboratorio de Alimentos, Centro de Investigación en Nutrición y Salud Pública, Facultad de Salud Pública y Nutrición, Universidad Autónoma de Nuevo León, Monterrey, Mexico

**Keywords:** broad beans, autoimmunity, immunonutrition, AhR, Tregs, dysbiosis, biotransformation

## Abstract

Legumes are associated with gut health benefits, and increasing evidence indicates that their consumption reduces the risk of chronic diseases that include autoimmunity. Beans are rich sources of compounds with health-promoting effects, and recent metabolomic approaches have enabled the comprehensive characterization of the chemical composition of *Vicia faba* L. This article reviewed whether the phytocompounds in broad beans might modulate the aryl hydrocarbon receptor (AhR), which plays an essential role in autoantigen tolerance as a potential dietary strategy for autoimmune disease management. Therefore, thirty molecules present in *Vicia faba* of the chemical classes of flavonoids, chalcones, stilbenes, jasmonates, alkaloids, and amino acids, and either a human- or microbiome-derived product of biotransformation, retrieved from the literature or predicted *in silico* were evaluated by docking for affinity against the ligand-binding domain of AhR. Most analyzed compounds showed high affinity even after their metabolism which indicate that some AhR modulators remain active despite several steps in their biotransformation. Hence, our results suggest that in similitude with the gut metabolism of the tryptophan, phytocompounds mainly polyphenols also lead to metabolites that induce the AhR pathway. Furthermore, wyerone acid, wyerone epoxide, jasmonic acid, stizolamine, vicine, and convicine and their metabolite derivatives are reported for the first time as potential AhR ligands. Overall, chronic consumption of phytochemicals in *Vicia faba* L. and their gut biotransformation may protect against autoimmune disease pathogenesis by AhR modulation.

## Introduction

The broad bean (*Vicia faba* L.) belongs to the Fabaceae family of plants characterized by the production of fruits called legumes. The genus *Vicia* contains about 140 species, and broad beans are the oldest seeds to be domesticated and the seventh-most produced legume worldwide with a yield of more than 3 million tons per year (1, 2). The nutritional value of broad beans per 100 g dry weight is composed of approximately 320 kilocalories distributed in 40% of protein, 4% lipids, and 56% carbohydrates. Hence, broad beans are recognized as a food with high content of protein, starch, and fiber with low levels of lipids; however, these values might vary depending on the seed variety or its degree of maturation (2, 3).

In traditional medicine *Vicia faba* leaves, roots, sprouts, pods, and beans have been used as infusion or food for the natural management of several chronic illnesses such as certain types of cancer, diabetes, cardiovascular diseases, anemia, malaria, depression (1), Parkinson's disease (4), allergies (5), diarrhea (6), and stomach ulcers (7). Many of these beneficial attributes are associated with the presence of non-nutritional compounds; in this regard, modern analytical hyphenated techniques have enabled the comprehensive characterization of more than 240 bioactive phytocompounds in these pulses (8, 9). A complex phytochemical profile is expected since *Vicia faba* possesses the largest genome in the legume family with 13.4 gigabase pairs (2). In addition, recent RNA-seq technology shows that the genome-wide transcriptome profile of broad bean seeds is composed of 15,697 expressed genes, and the most significantly enriched pathways are related to metabolism, followed by hormonal signal transduction, plant–pathogen interactions, and the biosynthesis of alkaloids, phenylalanine, tyrosine and tryptophan, flavonoids, and stilbenes (10). Furthermore, chemical characterization by high-performance liquid chromatography hyphenated to mass spectrometry is consistent with the transcriptomic studies since most of the identified metabolites belong mainly to flavonoids, chalcones, stilbenes, jasmonates, phenolic acids, and alkaloids (8, 9).

Bioactive compounds considered for this review were described in broad beans (8, 11–14). Phenolic compounds are the major class of secondary metabolites, with phenolic acids and polyphenols, which accounts for ~85%. Phenolic acids are mainly divided into hydroxycinnamic acids derived from cinnamic acid and hydroxybenzoic acids derived from benzoic acid. The most common hydroxycinnamic acids in broad beans are the ferulic, caffeic, coumaric, sinapic, and coutaric acids. Regarding the hydroxybenzoic acids, the most abundant are the protocatechuic, syringic, vanillic, gallic, and salicylic acids. The phenylpropanoic acids such as piscidic, fukiic, and eucomic are biosynthetically related to benzoic acids and also highly abundant in broad beans (8, 9). The presence of flavonoids and their derivatives is remarkable, being the most abundant flavonols such as quercetin, kaempferol, and myricetin, followed by the flavanols such as catechin and epicatechin. It also contains the flavone apigenin, naringenin, chrysin, geraldone, luteolin, and its methoxy derivative diosmetin, the flavanone eriodictyol, and the anthocyanin pelargonidin (8, 9). In addition, *Vicia faba* contain low-to-moderate levels of the isoflavones such as genistein, daidzein, formononetin, and the pterocarpan derivatives such as medicarpin and coumestrol (8, 9, 12). Also derived from the shikimate pathway (15), the chalcones, such as phloretin, butein (14), and the stilbene resveratrol (8, 9), and the aromatic amino acids, such as phenylalanine, L-tyrosine, and L-DOPA, are present in broad beans (13). This legume is considered as a good source of the essential amino acids such as arginine, phenylalanine, valine, threonine, and tryptophan; in contrast, methionine and cysteine are the limiting amino acids in the broad seeds (16). Legumes also contain several bioactive compounds that are traditionally classified as antinutrients, such as phytates, saponins, lectins, protease inhibitors, and alkaloids (11). Vicine and convicine (17) are pyrimidine alkaloids that were recently characterized to be derived from purine metabolism (18), whereas the origin of the pyrazine derivative stilozamine (19) remains to be elucidated. Finally, the jasmonic acid and its analogs have been recognized as the major components in *Vicia faba* phytoalexins and include the tuberonic acid, wyerone acid, and wyerone epoxide (20, 21).

The emerging role of the aryl hydrocarbon receptor (AhR) receptor pathway in the human gut for the generation of T regulatory cells (Tregs) (22) in the etiopathogenesis of autoimmune diseases (23–25) suggests that broad beans might benefit these types of diseases. According to current knowledge, the compounds present in *Vicia faba* that can activate the AhR include the quercetin, myricetin, kaempferol, chrysin, apigenin, naringenin, genistein, luteolin, phloretin, daidzein, diosmin (26), coumestrol (27), resveratrol (28), catechin, epicatechin (29), pelargonidin (30), tryptophan (31), and L-DOPA (32). Dietary AhR ligands have been successfully shown to induce immunomodulatory and antiinflammatory effects by influencing the phenotype of dendritic (24), T helper (33), B cells (34), and the cytokine microenvironment (35). Furthermore, flavonoids at dietary levels modulate the AhR *in vivo* (29), and part of the beneficial effects of phenolic compounds have been recently attributed to its regulation (30). Some studies have been addressed that indoles and alkaloids can attenuate experimental models of autoimmune diseases by the AhR-dependent induction of Tregs (33, 36). In addition, the AhR pathway is crucial in the sensing of dietary microbial-derived metabolites and for the generation of Tregs (37). The metabolites produced by commensal bacteria promote the induction of Tregs (38), and increasing evidence shows that autoimmune patients lack both, enough health-promoting bacteria and tolerogenic T cells (39–42).

This review explores the possibility that the chemical profile of *Vicia faba* beans promotes tolerogenic effects in autoimmune patients by AhR modulation. The interplay among diet, microbiota, and the mechanisms of Tregs generation is provided with a focus on its modulation *via* AhR ligands for autoimmune disease etiopathogenesis. The rationale behind the potential promotion of tolerogenic responses in the gut mediated by broad beans phytochemicals by the AhR pathway is discussed along with their traditional role as foods with high fiber content and prebiotic potential. The phytochemicals considered for the analysis were retrieved from the literature and crossreferenced with their ability to bind the AhR. In addition, it was assessed that their capacity to remain as AhR ligand despite biotransformation. For this purpose, one derived metabolite of each phytocompound was collected from reports, either from phase I, phase II, or biotransformed by the microbiome. When information was unavailable, a prediction *in silico* was carried out. The results of the docking of *Vicia faba* compounds and their derived metabolites are discussed considering current knowledge regarding the biotransformation of dietary phytocompounds by the host and microbiome and also their binding affinity for AhR after metabolism.

## Autoimmunity and The GUT

Autoimmune diseases are characterized by aberrant activation of the adaptative immune system against self-antigens (43). In systemic lupus erythematosus, rheumatoid arthritis, type 1 diabetes, psoriasis, multiple sclerosis, or Hashimoto's thyroiditis, the immune system ability to recognize self-components is higher than normal and associated with organ dysfunction or irreversible tissue injury mediated by autoantibodies or lymphocytes that led to clinical consequences (43). Although autoimmune diseases are heterogeneous and multifactorial, they may partially overlap, and their clinical course is chronic and often requires lifelong disease management (43).

Predisposing genetic risk factors account for autoimmune diseases onset; however, it is understood that plays a minor role in the current overall disease burden (44). Instead, nowadays, it is well-accepted that the etiology of many autoimmune diseases involves environmental factors. Among them, the consumption of western diets has been linked with the increasing incidence of these types of diseases. Furthermore, frequent eating of processed foods rich in salt, fat, protein, and sugar has been associated not only with an increasing prevalence of autoimmunity but also with an overall worst prognosis (45). The change of dietary habits has been under intensive investigation, which reveals a direct influence on immune homeostasis and bacterial communities colonizing the gastrointestinal tract (46–48). The mammalian intestine contains a complex symbiosis of epithelial and immune cells with commensal microorganisms (48) that in turn are heavily influenced by food components (45, 46).

The lymphoid tissue associated with the gut is the largest immune organ of the body. It forms a complex network of Peyer's patches, mesenteric lymph nodes, lymphoid follicles, intraepithelial, or lamina propria lymphocytes (49). They include different subpopulations of T cells, B cells, NK cells, and macrophages (49). The epithelial microfold cells capture diet and microbial-derived antigens from the Peyer's patches and facilitate the delivery of luminal antigens to dendritic cells and macrophages that migrate to lymphoid follicles and mesenteric lymph nodes, where those antigen-presenting cells expose and activate T cells that differentiate into effector cells (50). During the process of cell fate commitment, naïve T cells are influenced by the cytokine milieu, the microbiota composition, and luminal dietary metabolites. Hence, those factors shape the subset of T helper cells that are generated in the periphery (46, 51, 52).

The reduction in the levels of Tregs is associated with higher disease activity and poor prognosis in autoimmune patients. Therefore, the deficiency of these cells has been linked to the etiopathogenesis of autoimmunity. In addition, this phenomena also occur in the experimentally induced models of several autoimmune diseases (39). Tregs are a unique subpopulation of CD4+ T cells with properties in the maintenance of immune tolerance, thus preventing responses against food or commensal components and also avoiding reactivity against self-antigens. This subset of cells can inhibit the proliferation and activation of effector T cells by cell contact or secretion of TGF-β, IL-10, granzyme, and perforin (22). Another common finding in autoimmune patients is the alterations in the composition of gut microbiota that are suspected to be involved in disease etiopathogenesis (40–42). In normal conditions, the dominant gut microbial phyla are *Actinobacteria, Proteobacteria, Fusobacteria, Verrucomicrobia, Firmicutes*, and *Bacteroidetes*, with these last two representing 90% of gut microbiota. The *Firmicutes* are composed of more than 200 different genera such as *Lactobacillus, Bacillus, Enterococcus, Ruminicoccus*, and *Clostridium*, which this last accounting for 95% of the phylum. *Bacteroidetes* consist mainly of the genera *Bacteroides* and *Prevotella* whereas the phylum *Actinobacteria* is less abundant and represented by the *Bifidobacterium* genus (53). Patients with autoimmune diseases in general exhibit an increment of the taxa *Bacteroidetes* at the expense of the *Firmicutes* when compared to healthy subjects (40–42). In patients with lupus, *Rhodococcus, Eggerthella, Klebsiella, Prevotella, Eubacterium*, and *Flavonifractor* are significantly enriched, whereas *Dialister* and *Pseudobutyrivibrio* decreased (40). In rheumatoid arthritis, the gut microbiome was characterized by an increase of *Prevotella* and lower numbers of *Bifidobacteria, Bacteroides*, and *Clostridium* (54). Patients affected by multiple sclerosis display a decrease in *Bacteroides, Faecalibacterium* whereas *Methanobrevibacter, Enterobacteriaceae*, and *Akkermansia* showed an increment (42).

The disturbance in the balance of the microbiota and its reduction in complexity is also called dysbiosis and together with the diminution in the levels of Tregs that seem closely connected with the loss of tolerance to autoantigens (39, 52). Remarkably, the *Firmicutes* of the class *Clostridia* are reduced in autoimmune patients and these types of microorganisms demonstrate an important role in the induction of Tregs (41, 42). The mechanism underlying those effects is linked with the influence of commensal bacteria on the type of cytokine production by dendritic cells. Hence, it partially explains why lymphocyte differentiation is affected by the type of microbiota composition (55). For example, the presence of *Firmicutes*, such as *Lactobacillus*, is sensed by dendritic cells *via* toll-like receptors and induces the production of the regulatory cytokines such as interleukin 10 (IL-10) and transforming growth factor beta (TGF-β) that in turn promote the generation of Tregs (55). In the case of *Clostridia*, they promote the expansion and differentiation of Tregs by the breakdown of indigestible dietary components, such as fibers, and producing short-chain fatty acids (56). The acetate, propionate, and butyrate are microbiota-derived fermentation products that are important for intestinal epithelial cells as fuel. In addition, they affect their proliferation, differentiation, and gene expression that improve the gut barrier function. Furthermore, through the activation of the surface G protein-coupled receptors, they also induce antiinflammatory responses (57).

Recent discoveries have underscored that gut microbiota not only modulate the immune system through their antigens or fiber fermentation but also by the biotransformation of indoles. The most studied example is tryptophan and their products of metabolism that act as AhR ligands (37). Tryptophan is decarboxylated to tryptamine by the *Firmicutes Clostridium* and *Ruminococcus*. Derivatization of indole pyruvic acid from tryptophan is catalyzed by tryptophanase, and then, indole pyruvic acid is decarboxylated to indole acetaldehyde, which is the precursor of tryptophol and indole acetic acid. In turn, this is converted to 3-methyl indole by *Lactobacillus, Clostridium*, and *Bacteroides*. Indole pyruvic acid can also be converted to indole lactate, indole acrylic acid, and indole propionic acid by commensal microorganisms. Indole propionic acid can be further converted to indole acrylic acid in the liver or kidney and combined with glycine to produce indolyl acryloyl glycine. In addition, our epithelial intestinal cells produce tryptophan derivatives with AhR affinity. The tryptophan through 2,3-dioxygenase and indolamine 2,3-dioxygenase produce kynurenine; in turn, aminitric oxide transferase generates xanthurenic acid and kynurenine acid (58). Overall, microbial and endogenous biotransformation of dietary tryptophan in the gut can also induce Tregs, mucosal, and immune homeostasis by AhR modulation (59).

Studies show that it is possible to modify the intestinal microbiota with drugs, natural products, diet, probiotics, and prebiotics (60). In autoimmune patients, this is of utmost importance since dysbiosis plays a key role, and therapies used to control the symptoms such as antibiotics and methotrexate could negatively influence the microbiome composition (61, 62). There are also data to suggest that some specific types of antibiotics, probiotics, short-chain fatty acids, and fecal microbiota transplantation hold the potential to significantly improve autoimmune disease activity (60). The participation of the AhR in the interactions of dietary compounds with the microbiota and the lymphocyte responses that characterize autoimmune patients seems crucial since several plants or commensal-derived metabolites act as AhR ligands that induce the generation of Tregs (22, 36, 59, 63–65). The activation of the AhR pathway in the intestine by dietary or microbiome metabolites triggers immunoregulatory effects that have been recognized to be implicated in the pathogenesis of autoimmune disorders (23, 66, 67). Therefore, targeting the AhR signaling has been suggested as a promised approach for autoimmune disease management (23–25, 68).

## The Tolerogenic GUT, The AhR connection

The AhR protein is a ubiquitously expressed transcription factor involved in sensing environmental factors. It exists as a multiprotein complex with the proteins such as heat shock protein 90, the AhR-interacting protein, the chaperone p23, and the cellular-sarcoma protein kinase. The AhR transcription factor contains a basic helix–loop–helix domain involved in DNA binding and two Per-Arnt-Sim (PAS) domains. The PAS-A domain controls dimerization and enhances DNA binding, whereas the PAS-B domain contains several conserved residues critical for ligand binding. In addition, the C-terminal region of the AhR protein is located a glutamine-rich domain involved in coactivation and transactivation (69). Ligand binding releases the AhR-interacting protein from the complex and triggers conformational changes that expose its nuclear localization signal, which leads to its translocation. Once in the nucleus, the association of AhR with the AhR nuclear translocator results in the transcriptional control of multiple target genes, such as xenobiotic-metabolizing enzymes of the phase I and phase II, and transporters of the phase III metabolic pathways, which include the microsomal cytochrome P450-dependent monooxygenases CYP1A1, CYP1A2, 1, CYP1B1, the NAD(P)H-quinone oxidoreductase, the UDP-glucuronosyltransferase 1A1, and the multidrug resistance mutation 1 ATP-binding cassette transport protein (70). Therefore, it seems that AhR ligands induce their biotransformation and clearance from the body. Dietary, microbial, and many other environmental molecules can activate AhR, which suggests that ligand promiscuity allows the adaptation of cells to chemical challenges through this pathway (70).

The genetic control of the immune response mediated by the AhR remains to be fully clarified; however, its interaction with the *transcription factor nuclear factor-kappa light chain enhancer of activated B cells* (NF-κB) suggests direct functions as a coordinator of inflammatory and survival responses. AhR dimerizes with NF-κB subunits such as RelA and RelB, which leads to its recruitment to consensus DNA sequences and promoting gene activation. The AhR/Rel dimers can be found in lymphocytes where they bind DNA response elements of both transcription factors and support the activation of target genes of the AhR and the NF-κB pathways (71). Noticeably, different ligands have been suggested to induce the association of AhR with distinct proteins, thus potentially changing the type of gene expression in accordance with the stimulus (72).

The AhR pathway is a key for the homeostasis in the intestine and promotes epithelial renewal and barrier integrity along with immunomodulatory properties (69). Its activation in response to structurally diverse ligands alleviates autoimmune conditions by promoting the differentiation of Tregs and reducing proinflammatory mediators in animal models of inflammatory and autoimmune diseases (23–25, 30, 33, 36). The treatment of cultured naive T cells and dendritic cells with dietary AhR ligands results in an increase of the frequency of Tregs in a concentration-dependent manner (73). In addition, the induction of AhR signaling enhances de secretion of the antiinflammatory cytokines such as IL-22 and IL-10 while reducing the production of the proinflammatory cytokines such as tumor necrosis factor-alpha, IL-1, IL-6, and IL-12 that are also the desirable effects against autoimmunity (34, 35, 73).

Overall, the dietary administration of AhR ligands is a promising strategy for the management of autoimmune diseases since it positively influences the cytokine microenvironment (35) and the phenotype of dendritic (24), T helper (33), and B cells (34). Although the primary role of the diet is to provide nutrients to fulfill metabolic requirements, the use of foods to improve health and wellbeing is being increasingly accepted (74). Moreover, being that dysbiosis also contributes to autoimmunity (42) and diet can restore the microbiome, recent studies support the concept that foods can be viewed as a means to prevent immune-mediated diseases (46). Previously, broad beans have been proposed as an important tool to overcome diseases associated with gut dysbiosis. Along with other legumes such as black beans, cowpea seeds, lentils, chickpeas, and lupin seeds, the broad beans increase the production of short-chain fatty acids mediated by the proliferation of *Bifidobacterium* (75, 76). As previously reviewed, those effects alone are theoretically enough to improve the tolerance against autoantigens since both factors are essential in the promotion and differentiation of the Tregs. In the present contribution, it was suggested that the chemical profile *Vicia faba* beans promotes tolerogenic effects in autoimmune patients *via* AhR. To further support our proposal, the following provides a brief description of the metabolism and biotransformation of phytochemicals in general, along with a specific review of the metabolic fate of 30 phytocompounds present in broad beans and their capacity to bind the AhR before and after their human or microbial biotransformation. When data were not available in the current literature, results were predicted by *in silico* approaches.

## Exploring the metabolic fate of phytochemicals in broad beans and their AhR-Binding capacity

In general, most dietary phytochemicals are present in their glycosidic forms, that is, bond to one or more sugars through their phenolic or hydroxyl groups. Hence, the first step in metabolism is their deglycosylation in the brush border by associated enzymes of the intestine that hydrolyzes the compounds (77). Then, aglycons can be subjected to further metabolism by phase I reactions that usually produce more reactive lipophilic xenobiotics by adding or modifying functional groups, such as amino, hydroxy, or carboxyl groups. In Phase II, those metabolites are prepared for excretion by the addition of conjugated cofactors by reactions of glucuronidation, sulfation, methylation, and acetylation that make them less toxic and hydrophilic. Most of the biotransformation takes place within the liver and intestine; for example, following intestinal conjugation, absorbed flavonoids are transported to the portal vein, and, in the liver, multiple derivatives are produced (77). Then, metabolites can be excreted by enterocytes back into the intestinal lumen and discharged with the feces. However, metabolites are also deconjugated again by the microbiota and reabsorbed. Microbial enzymes eliminate glycosides, glucuronides, and sulfates and produce flavonoid aglycons increasing the half-life of dietary phytocompounds in the plasma (78). In addition, bacterial reactions are more complex and include deglycosylation, demethylation, dihydroxylation, ester cleavage, reduction of double bonds, isomerization, ring fission, and decarboxylation. In contrast to the hepatic metabolism of xenobiotics, the microbiota do not involve oxygen but rather reductions and hydrolysis, which results in non-polar low molecular weight products (79).

Unlike tryptophan and its derivatives, the metabolic fate of phytochemicals concerning their AhR-binding affinity remains largely unexplored. Therefore, an *in silico* binding analysis was carried out for thirty compounds and their metabolites against AhR. As no experimentally determined structures of the AhR ligand-binding domain are available and previous homology models were only derived from apo template structures, we follow two methods to predict the binding capacity of AhR ligands that were experimentally confirmed. The first report addressed the affinity of flavipin a flavonoid derivative against the Ahr PAS-A domain (80) and the second is based on holo X-ray structures of the hypoxia-inducible factor 2α (HIF-2α) PAS-B domain (81). The ligand capacity of the flavipin was validated by its ability to induce the expression of Ahr downstream genes in lymphocytes and to ameliorate autoimmune disease severity *in vivo* (80) whereas the *in silico* ability of HIF-2α crystallographic ligands also resulted in gene expression AhR-dependent *in vitro* (81). Of notice, the *in silico* binding results for flavipin were reproduced, and the simulations of the 30 compounds and their derivatives were carried out with the same parameters for the Ahr PAS-A domain. In brief, PDB files for molecular docking were collected from Protein Data Bank, the candidate-binding sites were predicted using the DoGSiteScorer, the 3D coordinates were obtained by molecular dynamics through Avogadro, and the molecular docking was performed in the rigid modality in AutoDock. The results of the simulation with the second method were consistent the first analysis; hence, for the sake of clarity, only the results for the Ahr PAS-A domain are presented in Table 1 (the full description of both methods, the complete results, binding energy, inhibition constant, and the formation of hydrogen bonds of each molecule including the simulations for the HIF-2α PAS-B domain are provided in Supplementary Material).

**Table 1:** Results of the simulations to assess the binding energy of compounds that characterized the chemical profile of *Vicia faba* beans against the PAS-A domain of the AhR before and after their human or microbiome biotransformation.

**Class**	**Molecule**	**Binding energy (kcal/mol)**	**Biotransformation by human enzymes or microbiome (Ref)**	**Derived metabolite**	**Binding energy (kcal/mol)**
Alkaloids	Stizolamine	−5.01	Cytochrome P450 (82)	(E)-3-(l-oxidaneyl)- 2-azaneyl l-azaneyl)methylene) amino)-6-((l-oxidaneyl)methyl)-1-methylpyrazin-2-(1H)-one	−5.76
	Vicine	−7.00	*Lactobacillus plantarum* (83)	Divicine	−4.99
	Convicine	−7.61	*Lactobacillus plantarum* (83)	Isouramil	−5.55
Aminoacids	L-Tryptophan	−5.62	*Ruminococcus gnavus* (84)	Tryptamine	−5.95
	L-DOPA	−5.36	*Eggerthella lenta* (85)	m-Tyramine	−5.81
	L-Cystine	−1.90	Cytochrome P450 (86)	L-Cysteine	−3.49
Anthocyanins	Pelargonidin	−7.03	*Lactobacillus plantarum* (87)	4-Hydroxybenzoic acid	−5.08
Chalcones	Butein	−6.86	Cytochrome P450 (82)	Neoplathymenin	−7.16
	Phloretin	−6.94	*Eubacterium ramulus* (88)	Phloretic acid	−5.23
Flavonoids	Coumestrol	−6.15	Cytochrome P450 (89)	8-Methoxycoumestrol	−5.40
	Luteolin	−6.97	*Clostridium orbiscindens* (90)	3-(3-Hydroxyphenyl)-propionic acid	−4.94
	Diosmetin	−7.37	*Escherichia coli* (91)	Citrifoliol	−7.50
	Daidzein	−7.49	*Slackia equolifaciens* (92)	Equol	−7.32
	Catechin	−7.36	*Flavonifractor plautii* (92)	5-(3',4'-Dihydroxyphenyl)-gamma-valerolactone	−6.75
	Chrysin	−6.48	*Blautia sp* (93)	Baicalein	−6.71
	Epicatechin	−7.36	*Eubacterium oxidoreducens* (94)	Phloroglucinol	−5.64
	Apigenin	−6.85	*Clostridium orbiscindens* (90)	4-Hydroxycinnamic acid	−5.53
	Gallocatechin	−7.63	*Eggerthella sp* (95)	1-(3,4,5-trihydroxyphenyl)-3-(2,4,6-trihydroxyphenyl) propan-2-ol	−6.93
	Eriodictyol	−7.23	*Clostridium butyricum* (96)	3-(3,4-Dihydroxyphenyl) propionic acid	−5.68
	Kaempferol	−6.72	*Clostridium orbiscindens* (90)	2-(4-Hydroxyphenyl) propionic acid	−5.30
	Genistein	−7.55	*Eubacterium ramulus* (92)	6'-Hydroxy-O-desmethylangolensin	−6.80
	Quercetin	−6.89	*Clostridium orbiscindens* (90)	Taxifolin	−7.40
	Myricetin	−7.16	*Clostridium orbiscindens* (90)	2-(3-Hydroxyphenyl) acetic acid	−6.10
	Naringenin	−7.13	*Clostridium orbiscindens* (90)	3-(4-Hydroxyphenyl) propionic acid	−5.14
	Epigallocathechin	−7.50	*Flavonifractor plautii* (92)	4-Hydroxy-5-(3,4,5-trihydroxyphenyl)valeric acid	−5.88
Jasmonates	Wyerone acid	−6.08	Cytochrome P450 (82)	(E)-1-(5-(E)-3-(l-oxidaneyl)-3-oxoprop-1-en-1-yl)furan-2-yl)-6-hydroxyhept-4-en-2-yn-1-one	−6.12
	Jasmonic acid	−4.85	Cytochrome P450 (82)	(2R,3R)-3-(2-(l-oxidaneyl)-2-oxoethyl)-2-((E)-4-hydroxypent-2-en-1-yl)cyclopentan-1-one	−5.98
	Tuberonic acid	−5.30	Cytochrome P450 (82)	(2S,3R)-3-(2-(l-oxidaneyl)-2-oxoethyl)-2-(3,4-dihydroxybutyl) cyclopentan-1-one	−4.32
	Wyerone epoxide	−5.93	Cytochrome P450 (82)	(2R,3R)-3-(2-(l-oxidaneyl)-2-oxoethyl)-2-((E)-4-hydroxypent-2-en-1-yl)cyclopentan-1-one	−6.03
Stilbenes	Resveratrol	−6.64	*Slackia equolifaciens* (97)	Lunularin	−6.76

The calculated binding energies for stizolamine, vicine, convicine, L-cystine, wyerone acid, jasmonic acid, tuberonic acid, and wyerone epoxide against the AhR are provided for the first time. In the case of the products of biotransformation, except for tryptamine (98), m-tyramine (99), equol (100), baicalein (101), and taxifolin (102), the rest of the molecules lack reports addressing this subject. For comparative purposes, Table 1 shows our calculated binding affinities for all molecules along with a reference of its biotransformation. In this regard, most derived compounds were retrieved from the literature, mainly the products of the microbiota, but the stizolamine, butein, wyerone epoxide, and the tuberonic, jasmonic, and wyerone acids were obtained using the generator of the structures of likely cytochrome P450 metabolites based on predicted sites of metabolism (82). Due to the reiterative nature of the intermediaries in the degradation of flavonoids (77), different metabolites were selected to cover a higher degree of chemical entities in the steps of their catabolic pathway.

Quercetin, tryptophan, and resveratrol are the prototypical AhR ligands that can modulate and promote their nuclear translocation (28, 37, 103), and flavipin has recently been shown to ameliorate autoimmunity *in vivo via* aryl receptor gene activation (80); hence, their calculated binding energies of −6.89, −5.62, −6.64, and −4.56 kcal/mol were considered as positive controls in our *in silico* screening. Other criteria acknowledge a molecule as a ligand with high affinity when its calculated binding energy is −4.00 kcal/mol or below (104). Hence, according to our results, only L-cystine and their metabolite derivative L-cysteine lack ligand potential against the AhR receptor. In the case of flavonoids, they are considered natural AhR ligands (65). Therefore, the calculated binding energies in the range of −6.15– −7.63 kcal/mol are expected outcomes. However, the estimated values for jasmonates in the range of −4.85 to −6.08 kcal/mol suggest a potential ligand ability of those compounds. Although no reports address this subject, jasmonates are lipophilic plant hormones and this result might be related to the AhR-binding affinity of the lipoxin A4, an endogenous lipophilic metabolite of the arachidonic acid (105). The alkaloids such as stizolamine, convicine, and vicine also excel with a calculated affinity value of −5.01, −7.61, and −7.00 kcal/mol, respectively. In this regard, it has been suggested that vicine is sensing as a xenobiotic that potentially activates AhR mediated responses (106). In the case of L-DOPA, it shows binding energy of −5.36 kcal/mol, which is relatively close to the archetypal AhR ligand tryptophan. In addition, L-DOPA has been recently described as an AhR agonist *in vitro* (32), and broad beans are the richest food source of this compound (13). Current metabolomic approaches to food consumption consider the detection of vicine, convicine, wyerone acid, and wyerone epoxide as the biomarkers of broad beans (107). Therefore, although the most recognized natural AhR ligands, the flavonoids, along with the chalcones, anthocyanins, and stilbenes are widely distributed in the plant kingdom, the set of characteristic molecules in broad beans also hold potential as the modulators of the AhR.

**Figure 1:**
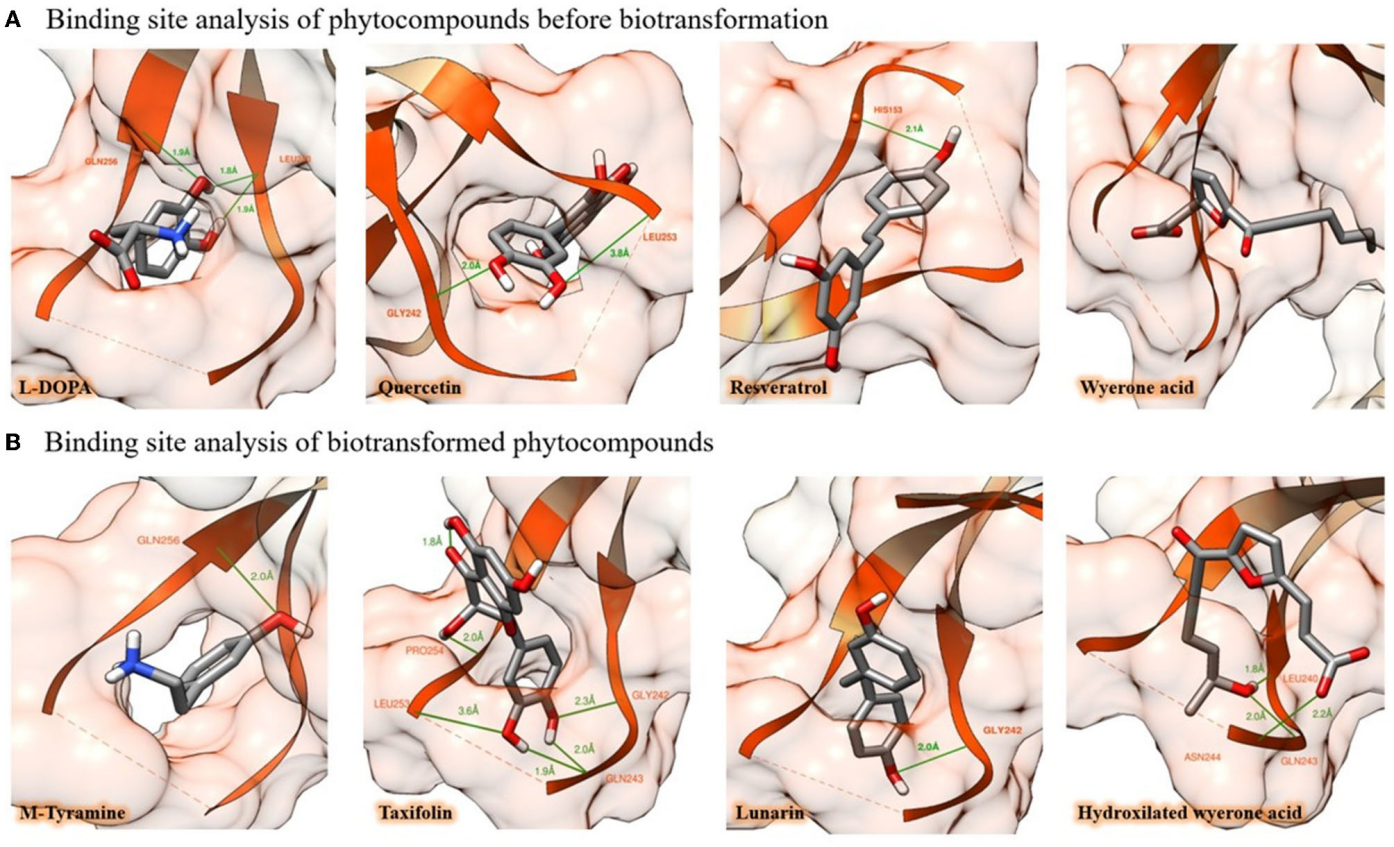
*In silico* binding potential of some natural products in broad beans against the PAS-A domain of the AhR. **(A)** Putative binding mode of L-DOPA, quercetin, resveratrol, and wyerone acid, to AhR protein, amino acid residues are highlighted in orange, whereas H-bond interactions of molecules are depicted with green lines. **(B)** After being biotransformed by enterocytes or microbiome, the natural products undergo chemical modifications that improve their binding capacity. In this figure, this process is illustrated by showing four events of biotransformation that resulted in enhanced AhR-binding affinity (the simulations of the 30 molecules, their figures, and binding energy before and after their biotransformation are provided in the Supplementary Material).

Regarding the metabolic fate of the selected compounds, the AhR-binding ability of tryptamine (98), an L-tryptophan microbiome derivative (84) was calculated as −5.95 kcal/mol. Hence, it is also consistent with the literature and highlights the reliability of the implemented method. An interesting finding of our *in silico* screening was that the biotransformation of L-tryptophan by *Ruminococcus gnavus* (84) results in a higher affinity for AhR. The same trend was observed for a biotransformation product of diosmetin, quercetin, genistein, resveratrol, and chrysin by the microbiota members *Escherichia coli* (91)*, Clostridium orbiscindens* (90)*, Eubacterium ramulus* (92), *Slackia equolifaciens* (97), and *Blautia sp*. (93), respectively. In the case of molecules considered the biomarkers of broad beans (107), the predicted cytochrome P450 metabolites for wyerone acid, wyerone epoxide, jasmonic acid, and stizolamine also show this effect. In addition, the biotransformation of L-DOPA by *Eggerthella lenta* to m-tyramine (85) and convicine to isouramil by the metabolic activities of *Lactobacillus plantarum* (83) also indicates the generation of ligands with increased affinity for the AhR (Figure 1).

Overall, our review of the metabolic profile of *Vicia faba* beans along with our *in silico* approach for assessing their AhR ligand potential before and after a reported or predicted metabolism by human or microbiota enzymes suggests that broad bean metabolites possess structural qualities to act as AhR modulators. In addition, our screening indicates that chalcones, flavonoids, stilbenes, jasmonates, anthocyanins, alkaloids, and aromatic amino acids are structurally suitable to act as AhR ligands even after biotransformation. Moreover, to our knowledge, this is the first report which suggests a potential role for alkaloids and jasmonates present in broad beans as AhR modulators.

The AhR signaling pathway is a prototypical link between the environment and the immune responses. According to the mechanism reviewed for the generation of Tregs mediated by dietary AhR ligands or derived from commensal bacteria, it is plausible that consumption of foods rich in molecules that modulate the AhR results in tolerogenic effects in the gut.

The potential of food to restore gut bacteria balance or to provide AhR ligands for the therapeutic management of autoimmune diseases has been previously discussed (25, 48, 76). Furthermore, it is well-described that flavonoids at levels achieved through feeding can activate the AhR (29), and that many health-promoting effects derived from dietary polyphenols are mediated by this pathway (30). In addition, it has been suggested that AhR occupancy and the persistence of modulators are more relevant than the dosage (108). However, many aspects of the AhR signaling remain to be elucidated since it is stimulated by a wide variety of chemical entities and forms transcriptional complexes with several proteins, which results in different patterns of gene expression (108).

**Figure 2:**
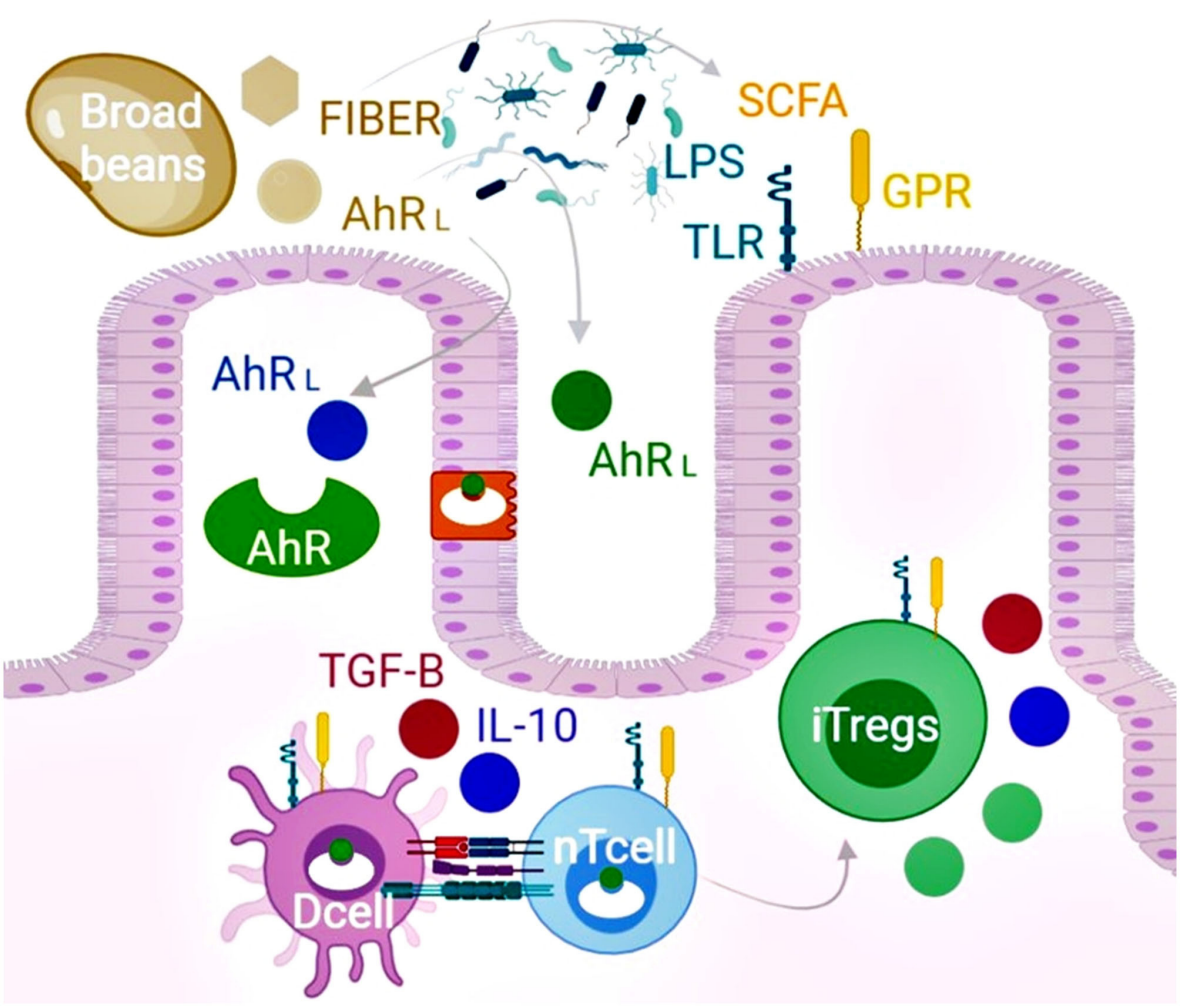
The chemical composition of broad beans promotes induced T regulatory cells (iTregs) and improves microbiota dysbiosis. Broad beans are foods rich in flavonols, flavanols, isoflavones, chalcones, stilbenoids, jasmonates, alkaloids, and L-DOPA that structurally act as the ligands of the AhR_L_. Upon binding, the receptor is translocated into the nucleus affecting the gene expression of several cell types that include intestinal epithelial cells, dendritic cells (Dcell), and naïve T cells (nTcell). The expansion of iTregs can inhibit autoreactive effector T cells by cell contacts and the secretion of TGF-β, IL-10, granzyme, and perforin. In addition, broad beans restore dysbiosis and increase the production of short-chain fatty acids and microbiota components (LPS) to promote the expansion of Tregs mediated by the activation of the surface G protein (GPR) and toll-like receptors (TLR). Furthermore, according to our *in silico* screening, the epithelial and microbiome biotransformation of the evaluated phytochemicals present in broad beans leads to more AhR ligands. Overall, consumption of broad beans might benefit autoimmune patients beyond their nutritional value *via* the AhR pathway and bifidogenic effects.

At present, no clinical interventions with broad beans have been carried out for autoimmune patients. However, diets that comprised mainly of legumes, vegetables, fruits, and grains reduce symptoms, biomarkers of disease severity and demonstrate overall benefits for autoimmune patients (109). Regarding the potential hematotoxicity by broad bean consumption, it seems very unlikely in autoimmune patients since favism is rare in adults (110). However, the relationship between diet and autoimmunity is complex, and it predicts whether broad beans might trigger adverse reactions in some autoimmune patients is challenging. On the other hand, it seems safe to speculate that their bifidogenic effect (75) itself might result in a potential net benefit for autoimmune patients. Here, we attempt to describe the complex interplay between diet, microbiota, and the immune system for the generation of self-tolerance, mediated mainly by the induction of Tregs through AhR ligands and the potential of targeting this axis by dietary interventions with a focus on the etiopathogenesis of autoimmune diseases and the chemical composition of broad beans (Figure 2). Most analyzed phytochemicals show structural qualities that make them natural AhR ligands despite gut biotransformation; hence, broad bean consumption might benefit autoimmune patients beyond its nutritional value and bifidogenic effects.

## Conclusion

According to the hypothesis, a diminution of dietary AhR ligands potentially underlies the low levels of Tregs and the loss of self-tolerance that leads to autoimmune diseases, hence, seems plausible that consumption of foods with high content of those type of phytocompounds may counteract their etiopathogenesis or aid in their management. Our *in silico* screening of molecules present in broad beans suggests that it contains chemical entities with AhR-binding capability that upon biotransformation may also preserve or increase their affinity. Of notice, this trend was also observed for the wyerone acid, wyerone epoxide, jasmonic acid, and stizolamine that not only are more confined to broad beans but also it is the first time that those compounds are proposed as AhR modulators. Hence, in addition to the reported bifidogenic effect for legumes, it is plausible that *Vicia faba* phytocompounds promote tolerogenic effects *in vivo via* AhR modulation with potential benefits for autoimmune patients.

## Author Contributions

LM-L and JV-R collected the information, interpreted, analyzed the data, wrote the manuscript, with contributions from DS, BG-M, and ML-C. LM-L establishes the proposal and provided expert knowledge in phytochemistry, immunobiology, and immunonutrition. DS performed the *in silico* analysis. BG-M and ML-C helped with funding. All authors read and checked the manuscript properly before submission.

## Funding

This work was supported by PAICYT (Programa de Apoyo a la Investigación Científica y Tecnológica) from Universidad Autónoma de Nuevo León (UANL).

## Conflict of Interest

The authors declare that the research was conducted in the absence of any commercial or financial relationships that could be construed as a potential conflict of interest.

## Publisher's Note

All claims expressed in this article are solely those of the authors and do not necessarily represent those of their affiliated organizations, or those of the publisher, the editors and the reviewers. Any product that may be evaluated in this article, or claim that may be made by its manufacturer, is not guaranteed or endorsed by the publisher.
