# Supplementary material for: Phytochemicals From Vicia faba Beans as Ligands of the Aryl Hydrocarbon Receptor to Regulate Autoimmune Diseases
Source: Front Nutr. 2022 Mar 4;9:790440. doi: 10.3389/fnut.2022.790440 (PMC8931403; doi:10.3389/fnut.2022.790440)
Supplement: Supplementary file 1 [file Data_Sheet_1.PDF]

Phytochemicals from *Vicia faba* Beans as Ligands of the Aryl Hydrocarbon Receptor to Regulate Autoimmune Diseases

Méndez-López, LF[1*], Sosa de León, D[1], López-Cabanillas Lomelí, M[1], González-Martínez, BE[1], Vázquez-Rodríguez, JA[1*].

[1]Laboratorio de Alimentos, Centro de Investigación en Nutrición y Salud Pública, Facultad de Salud Pública y Nutrición, Universidad Autónoma de Nuevo León, Monterrey, México.

* Correspondence:
Dr. Jesús Alberto Vázquez-Rodríguez, jesus.vazquezrdz@uanl.edu.mx
Dr. Luis Fernando Méndez-López, luis.mendezlop@uanl.edu.mx

Supplementary Material

1. *In silico* metabolism prediction using GLORY
2. *In silico* binding analysis against the aryl hydrocarbon receptor
3. Results of Docking
4. Binding mode analysis of AhR-ligand complexes

1. *In silico* metabolism prediction using GLORY

GLORY predict metabolites that can be formed in humans by enzymes belonging to the cytochrome P450 (CYP) enzyme family. Using a machine learning-based tool it predicts the metabolically labile atom positions in a molecule for CYP metabolism in humans. The tool was shown to have a high level of accuracy, achieving a Matthews correlation coefficient of 0.57 and an area under the curve of 0.91 on an independent test set. The reaction rules were developed based on known CYP-mediated reactions documented in the scientific literature. Hence the reaction rule base is not biased by any particular dataset.

The biotransformation of the following metabolites was obtained by GLORY tool since their metabolism remains unknown.

Table 1. Prediction of metabolites using GLORY.

Compound	Predicted Metabolite
Stizolamine	(E)-3-(l-oxidaneyl)- 2-azaneyl 1-azaneyl)methylene)amino)-6-((1-oxidaneyl)methyl)-1-methylpyrazin-2-(1H)-one
Wyerone acid	(E)-1-(5-(E)-3-(1-oxidaneyl)-3-oxoprop-1-en-1-yl)furan-2-yl)-6-hydroxyhept-4-en-2-yn-1-one
Jasmonic acid	2R,3R)-3-(2-(l-oxidaneyl)-2-oxoethyl)-2-((E)-4-hydroxypent-2-en-1-yl)cyclopentan-1-one
Tuberonic acid	(2S,3R)-3-(2-(l-oxidaneyl)-2-oxoethyl)-2-(3,4-dihydroxybutyl)cyclopentan-1-one
Wyerone epoxide	Methyl(E)-3-(5-(4-hydroxy-5-oxohept-2-ynoyl)furan-2-yl)acrylate

Reference

de Bruyn Kops, C., Stork, C., Šícho, M., Kochev, N., Svozil, D., Jeliazkova, N., & Kirchmair, J. (2019). GLORY: generator of the structures of likely cytochrome P450 metabolites based on predicted sites of metabolism. Frontiers in chemistry, 7, 402.

2. *In silico* binding analysis against the aryl hydrocarbon receptor

The *in silico* analysis began by collecting the PDB files needed for molecular docking from Protein Data Bank (www.rcsb.org). By employing the term "AhR", 7 structures were retrieved but only the file 4M4X was selected for docking based on its identity (PAS-A domain of AhR), PDB validation parameters and its MolProbity results. In the case of PAS-B domain, there were not crystallized files of AhR PAS-B domain. For this reason, the file 3H82 was selected from PDB due to some reports about homology of PAS-B domain between HIF-2α and AhR.

In order to perform the simulations, the candidate binding sites were predicted using the DoGSiteScorer tool from the ProteinPlus portal (www.proteins.plus). According with the drug score (Table 2), two sites were identified as the best candidates for binding simulation in PAS-A domain but only pocket 1 was considered for docking (Figure 1). For PAS-B domain, only one pocket was highlighted as the best site for docking (Figure 2). Indeed, this pocket agreed with the validation of HIF-2α PAS-B domain reported by Motto et al. (2011).

The 3D coordinates from ligands and their metabolites were obtained by molecular dynamics through Avogadro. Once the structures were ready, the molecular docking was performed in the rigid modality in AutoDock 4.2. The dimensions and 3D coordinates of the GridBox used in the simulations are shown in Table 3.

Once simulations were completed, binding energies, inhibition constants, and hydrogen bonds number were collected for each ligand in PAS-A and PAS-B domains. As crystallographic data about AhR is lacking, validation of molecular docking employing the redocking method is not possible. However, the predicted pockets used here are similar to those obtained by Hanieh et al. (2016). Indeed, our simulation of binding for flavipin against AhR PAS-A domain was in agreement with their prediction (-4.56 vs -4.63). Table 4 and Figure 3 contain the full results of the positive control.

Table 2. Characterization of predicted pockets for AHR using DoGSiteScorer.

AhR region	Pocket	Aminoacid residues in the site	Drug score
PAS-A	0	G107, L110, Q111, G113, E114, L116, L117, V126, F134, Y135, Q148, I152, L240 and F260	0.53
	1	T128, D130, L132, V133, F134, I152, H153, L240, H241, G242, Q243, N244, L253, P254, P255 and Q256	0.55
PAS-B	0	F244, S246, H248, S249, M252, F254, I261, L272, S276, A277, F280, Y281, D285, N288, M289, T290, S292, H293, L296, V302, V303, S304, G305, Q306, Y307, M309, L319, T321, Q322, G323, I337, C339 and N341	0.77

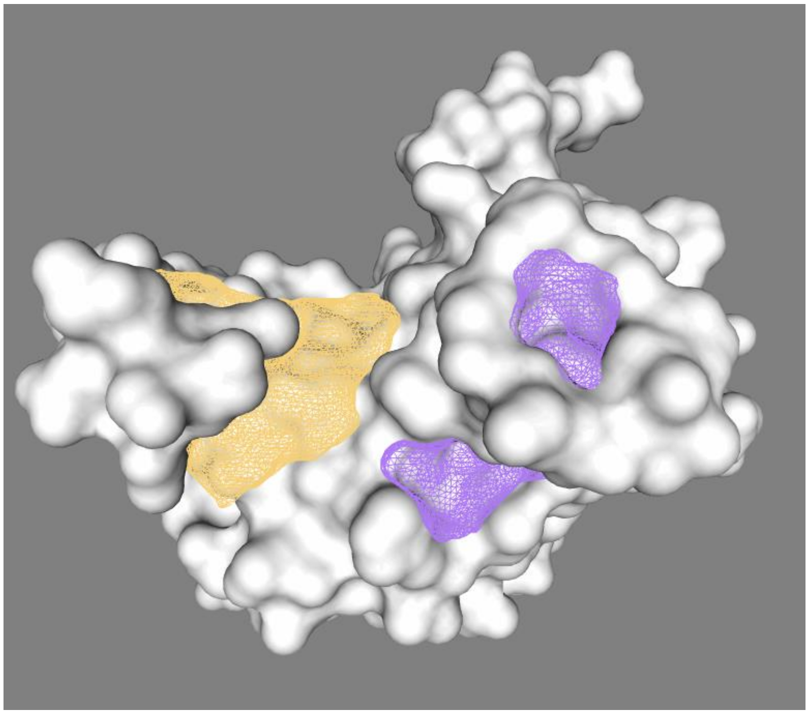

Figure 1. Predicted pockets for AHR PAS-A using DoGSiteScorer. Pocket 0 and pocket 1 are shown in yellow and purple, respectively.

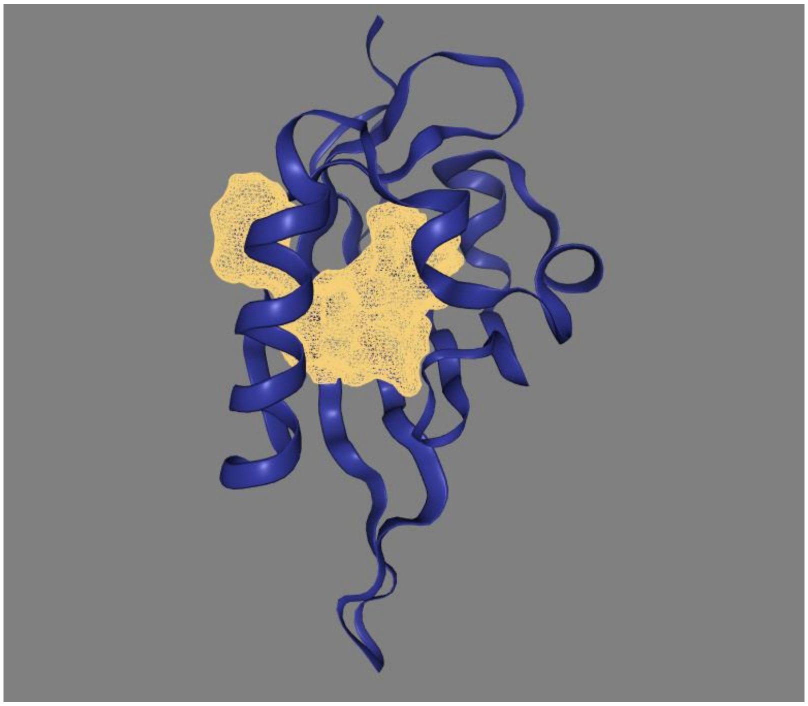

Figure 2. Predicted pocket for AHR PAS-B domain using DoGSiteScorer. Pocket 0 is shown in yellow.

Table 3. The dimensions and 3D coordinates of the GridBox used in the simulations

AhR Region	3D coordinates	Pocket 1
PAS-A	X-Dimension	54
	Y-Dimension	40
	Z-Dimension	50
	X-Center	38.769
	Y-Center	-9.246
	Z-Center	10.556
	Spacing (Angstrom)	0.375
PAS-B	X-Dimension	42
	Y-Dimension	34
	Z-Dimension	38
	X-Center	8.611
	Y-Center	-10.164
	Z-Center	5.034
	Spacing (Angstrom)	0.375

Table 4. Binding energy, inhibition constant, and hydrogen bonds resulted for the simulations of the flavipin against the AhR PAS-A domain in AutoDock 4.2.

Molecule	Binding Energy (kcal/mol)	Inhibition constant	Hydrogen bonding
Flavipin	-4.56	454.98 uM	4

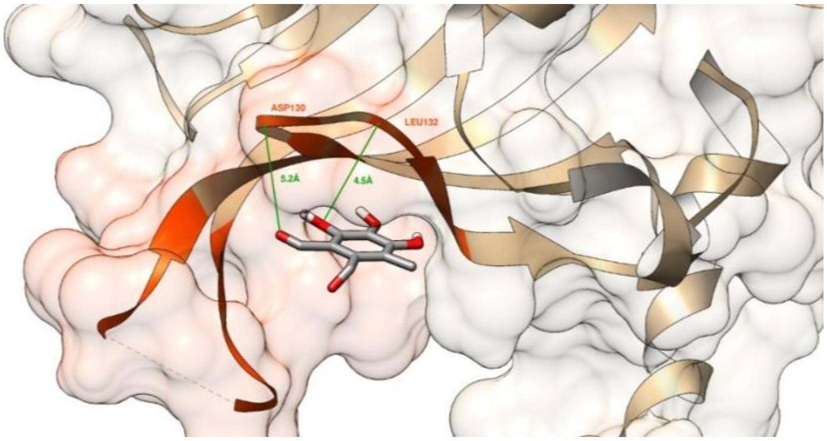

Figure 3. Binding mode analysis of flavipin in the pocket 1 of AhR PAS-A domain.

References

Morris, G. M., Huey, R., Lindstrom, W., Sanner, M. F., Belew, R. K., Goodsell, D. S., & Olson, A. J. (2009). AutoDock4 and AutoDockTools4: Automated docking with selective receptor flexibility. Journal of computational chemistry, 30(16), 2785-2791.

Hanwell, M. D., Curtis, D. E., Lonie, D. C., Vandermeersch, T., Zurek, E., & Hutchison, G. R. (2012). Avogadro: an advanced semantic chemical editor, visualization, and analysis platform. Journal of cheminformatics, 4(1), 1-17.

Volkamer, A., Kuhn, D., Grombacher, T., Rippmann, F., & Rarey, M. (2012). Combining global and local measures for structure-based druggability predictions. Journal of chemical information and modeling, 52(2), 360-372.

Hanieh, H., Mohafez, O., Hairul-Islam, V. I., Alzahrani, A., Bani Ismail, M., & Thirugnanasambantham, K. (2016). Novel aryl hydrocarbon receptor agonist suppresses migration and invasion of breast cancer cells. *PloS one*, *11*(12), e0167650.

Motto, I., Bordogna, A., Soshilov, A. A., Denison, M. S., & Bonati, L. (2011). New aryl hydrocarbon receptor homology model targeted to improve docking reliability. Journal of chemical information and modeling, 51(11), 2868-2881.

3. Results of Docking

Results of the binding energies, inhibition constants, and hydrogen bonds obtained from the simulations of the screening of 30 molecules in broad beans against the AhR.

Table 4. Molecular docking results for the interaction between constituents of broad beans and AhR.

Chemical Class	Molecule	PAS-A			PAS-B		
		Binding Energy (kcal/mol)	Inhibition constant	Hydrogen bonding	Binding Energy (kcal/mol)	Inhibition constant	Hydrogen bonding
Alkaloid	Vicine	-7.0	7.43 µM	6	-4.99	221.6 µM	2
Alkaloid	Stizolamine	-5.01	212.96 µM	2	-4.86	273.56 µM	2
Alkaloid	Convicine	-7.61	2.65 µM	4	-4.55	465.2 µM	3
Aminoacid	L- tryptophan	-5.62	75.83 µM	2	-5.93	45.25 µM	0
Aminoacid	L-DOPA	-5.36	117.57 µM	4	-4.45	550.06 µM	2
Aminoacid	L-Cystine	-1.90	40.73 mM	3	-3.31	3.76 µM	1
Anthocyanin	Pelargonidin	-7.03	7.02 µM	3	-6.78	10.79 µM	0
Chalcone	Butein	-6.86	9.3 µM	4	-7.36	4.05 µM	4
Chalcone	Phloretin	-6.94	8.25 µM	5	-7.42	3.64 µM	3
Flavonoid	Gallocatechin	-7.63	2.54 µM	5	-5.45	101.02 µM	3
Flavonoid	Genistein	-7.55	2.93 µM	1	-6.99	7.5 µM	0
Flavonoid	Epigallocathechin	-7.50	3.19 µM	5	-6.14	31.72 µM	2
Flavonoid	Daidzein	-7.49	3.23 µM	2	-7.28	4.62 µM	0
Flavonoid	Diosmetin	-7.37	3.95 µM	3	-7.12	6.03 µM	2
Flavonoid	Epicatechin	-7.36	4.05 µM	5	-6.13	32.27 µM	2
Flavonoid	Catechin	-7.36	4.01 µM	5	-6.13	32.12 µM	2
Flavonoid	Eriodictyol	-7.23	5.01 µM	2	-7.4	3.76 µM	3
Flavonoid	Myricetin	-7.16	5.67 µM	3	-6.57	15.32 µM	1
Flavonoid	Naringenin	-7.13	5.97 µM	2	-7.35	4.12 µM	1
Flavonoid	Luteolin	-6.97	7.8 µM	2	-7.32	4.34 µM	3
Flavonoid	Quercetin	-6.89	8.92 µM	2	-6.65	13.44 µM	0
Flavonoid	Apigenin	-6.85	9.6 µM	2	-7.37	3.94 µM	1
Flavonoid	Kaempferol	-6.72	11.91 µM	2	-6.65	13.36 µM	0
Flavonoid	Chrysin	-6.48	17.78 µM	1	-7.95	1.49 µM	1
Flavonoid	Coumestrol	-6.15	30.84 µM	4	-7.31	4.41 µM	1
Jasmonate	Wyerone acid	-6.08	34.9 µM	1	-6.09	34.47 µM	0
Jasmonate	Wyerone epoxide	-5.93	44.7 µM	4	-7.1	6.24 µM	2
Jasmonate	Jasmonic acid	-4.85	276.38 µM	1	-6.48	17.77 µM	2
Jasmonate	Tuberonic acid	-5.3	129.42 µM	2	-6.06	35.94 µM	2
Stilbene	Resveratrol	-6.64	13.49 µM	2	-6.81	10.13 µM	1

Results of the binding energies, inhibition constants, and hydrogen bonds obtained from the simulations of the screening of the biotransformed 30 molecules in broad beans against the AhR.

Table 5. Molecular docking results for the interaction between broad beans constituents-derived metabolites and AhR.

Class	Molecule	Derived Metabolite	PAS-A			PAS-B		
			Binding Energy (kcal/mol)	Inhibition constant	Hydrogen bonding	Binding Energy (kcal/mol)	Inhibition constant	Hydrogen bonding
Alkaloid	Vicine	Divicine	-4.99	220.77 µM	3	-3.52	2.63 mM	3
Alkaloid	Stizolamine	(E)-3-(l-oxidaneyl)- 2-azaneyl l-azaneyl)methylene) amino)-6-((l-oxidaneyl)methyl)-1-methylpyrazin-2-(1H)-one	-5.76	59.57 µM	2	-5.83	52.98 µM	4
Alkaloid	Convicine	Isouramil	-5.55	85.82 µM	4	-3.64	2.13 mM	2
Aminoacid	L- tryptophan	Tryptamine	-5.95	43.44 µM	1	-5.67	69.95 µM	3
Aminoacid	L-Dopa	m-Tyramine	-5.81	54.72 µM	2	-5.08	187.45 µM	3
Aminoacid	L-Cystine	L-Cysteine	-3.49	2.76 mM	3	-3.22	4.33 mM	1
Anthocyanin	Pelargonidin	4-Hydroxybenzoic acid	-5.08	187.91 µM	1	-4.58	439.47 µM	1
Chalcone	Butein	Neoplathymenin	-7.16	5.66 µM	3	-6.98	7.68 µM	0
Chalcone	Phloretin	Phloretic acid	-5.23	147.57 µM	1	-4.91	252.43 µM	1
Flavonoid	Gallocatechin	1-(3,4,5-trihydroxyphenyl)-3-(2,4,6-trihydroxyphenyl) propan-2-ol	-6.93	8.33 µM	3	-7.25	4.85 µM	4
Flavonoid	Genistein	6'-Hydroxy-O-desmethylangolensin	-6.80	10.31 µM	2	-7.0	7.42 µM	3
Flavonoid	Epigallocathechin	4-Hydroxy-5-(3,4,5-trihydroxyphenyl) valeric acid	-5.88	49.04 µM	6	-4.83	287.17 µM	3
Flavonoid	Daidzein	(S)-Equol	-7.32	4.28 µM	2	-6.98	7.7 µM	0
Flavonoid	Diosmetin	5,7-Dihydroxy-4′-methoxyflavone	-7.50	3.2 µM	2	-7.17	5.55 µM	1
Flavonoid	Epicatechin	Phloroglucinol	-5.64	73.07 µM	5	-4.16	900 µM	3
Flavonoid	Catechin	5-(3',4'-Dihydroxyphenyl)-gamma-valerolactone	-6.75	11.34 µM	4	-6.0	40 µM	1
Flavonoid	Eriodictyol	3-(3,4-Dihydroxyphenyl) propionic acid	-5.68	68.27 µM	3	-4.43	564.69 µM	1
Flavonoid	Myricetin	2-(3-Hydroxyphenyl) acetic acid	-6.10	33.73 µM	3	-5.07	191.41 µM	3
Flavonoid	Naringenin	3-(4-Hydroxyphenyl) propionic acid	-5.14	171.9 µM	1	-5.07	191.26 µM	2
Flavonoid	Luteolin	3-(3-hydroxyphenyl)-propionic acid	-5.40	101.26 µM	3	-5.18	158.48 µM	3
Flavonoid	Quercetin	Taxifolin	-7.40	68.27 µM	3	-6.86	9.39 µM	3
Flavonoid	Apigenin	4-hydroxycinnamic acid	-5.53	88.03 µM	1	-4.82	290.9 µM	1
Flavonoid	Kaempferol	2-(4-Hydroxyphenyl) propionic acid	-5.30	130.89 µM	1	-4.98	223.55 µM	0
Flavonoid	Chrysin	Baicalein	-6.71	11.98 µM	2	-7.72	2.2 µM	1
Flavonoid	Coumestrol	8-Methoxycoumestrol	-6.15	33.96 µM	3	-7.04	6.97 µM	0
Jasmonate	Wyerone acid	(E)-1-(5-(E)-3-(l-oxidaneyl)-3-oxoprop-1-	-6.12	32.86 µM	4	-6.28	25.15 µM	1

		en-1-yl) furan-2-yl)-6-hydroxyhept-4-en-2-yn-1-one						
Jasmonate	Wyerone epoxide	Methyl(E)-3-(5-(4-hydroxy-5-oxohept-2-ynoyl)furan-2-yl)acrylate	-6.03	37.93 µM	3	-7.57	2.85 µM	2
Jasmonate	Jasmonic acid	(2R,3R)-3-(2-(l-oxidaneyl)-2-oxoethyl)-2-((E)-4-hydroxypent-2-en-1-yl)cyclopentan-1-one	-5.98	41.34 µM	1	-6.26	25.91 µM	3
Jasmonate	Tuberonic acid	(2S,3R)-3-(2-(l-oxidaneyl)-2-oxoethyl)-2-(3,4-dihydroxybutyl) cyclopentan-1-one	-4.32	682.15 µM	4	-5.81	54.9 µM	4
Stilbene	Resveratrol	Lunularin	-6.76	11.14 µM	3	-7.07	6.56 µM	2

4. Binding mode analysis of AhR-ligand complexes

Binding mode analysis for interactions between the different ligands and AhR was performed employing UCSF Chimera 1.15. Hydrogen bonds were detected using the tool FindHBond with relax constraints parameters of 3 angstroms and 20 degrees. In the next figures, we showed the binding of the different ligands docked in pocket 1 of PAS-A domain. Those aminoacid residues that belong to pocket 1 are highlighted in orange. The hydrogen bonds predicted were highlighted in green lines with their corresponding distance in angstroms (Å).

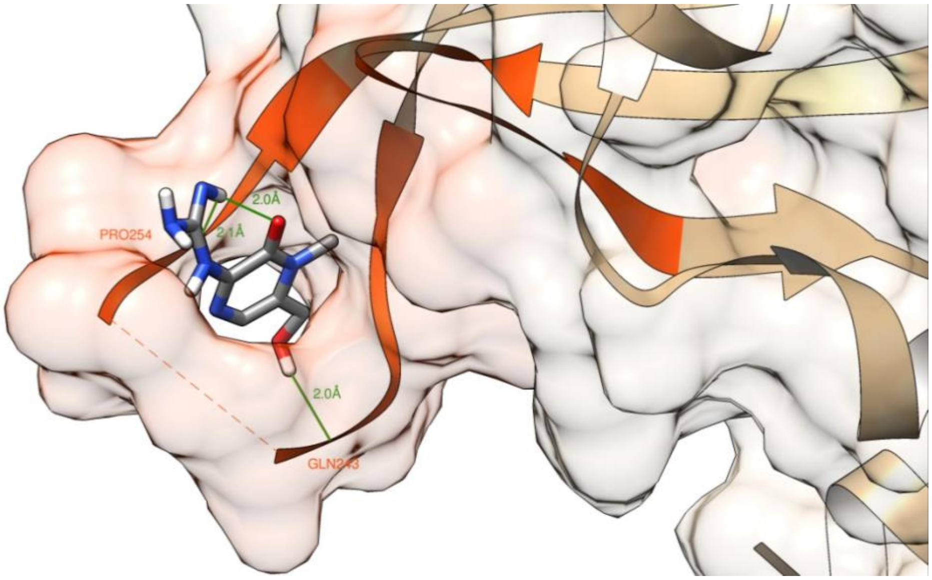

Figure 4. Binding mode analysis of stizolamine in the pocket 1 of AhR PAS-A domain.

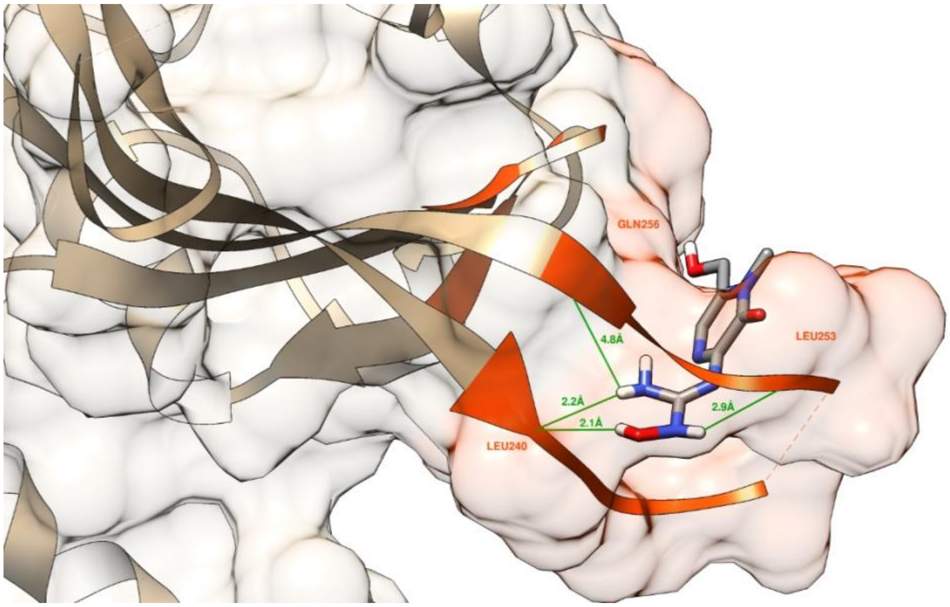

Figure 5. Binding mode analysis of (E)-1-hydroxy-2-(5-(hydroxymethyl)-4-methyl-3-oxo-3,4-dihydropyrazin-2-yl)guanidine (a metabolite derived from metabolism of stizolamine) in the pocket 1 of AhR PAS-A domain.

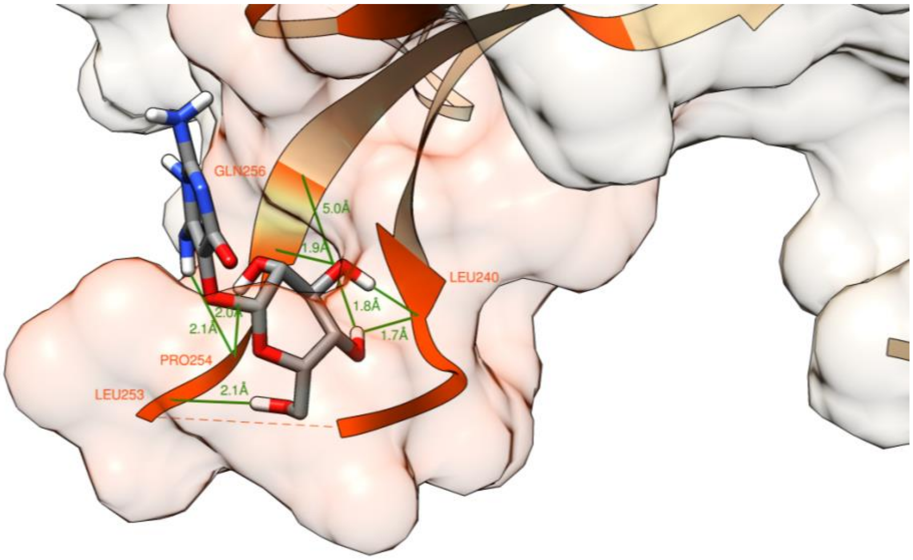

Figure 6. Binding mode analysis of vicine in the pocket 1 of AhR PAS-A domain.

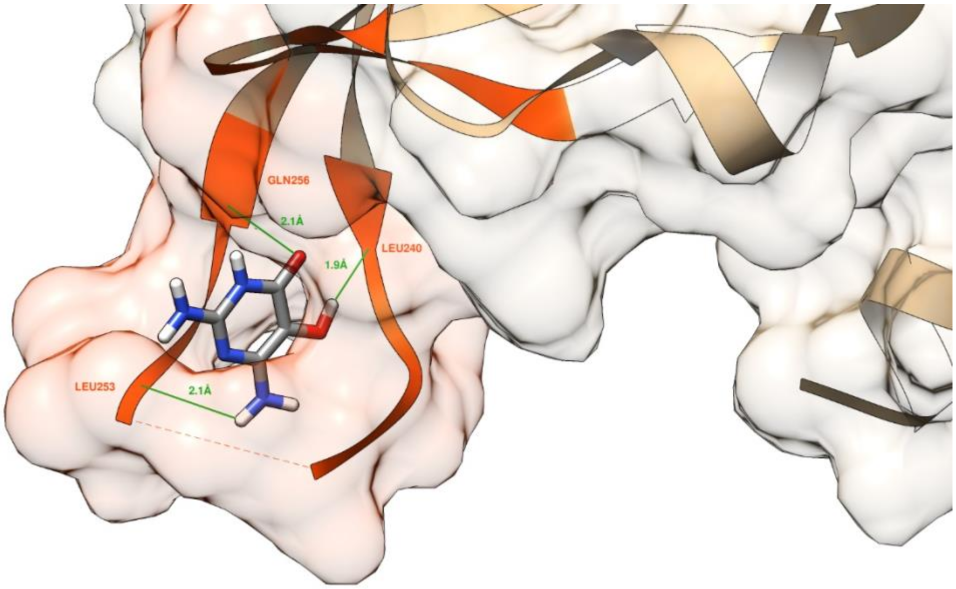

Figure 7. Binding mode analysis of divicine (a metabolite derived from metabolism of vicine) in the pocket 1 of AhR PAS-A domain.

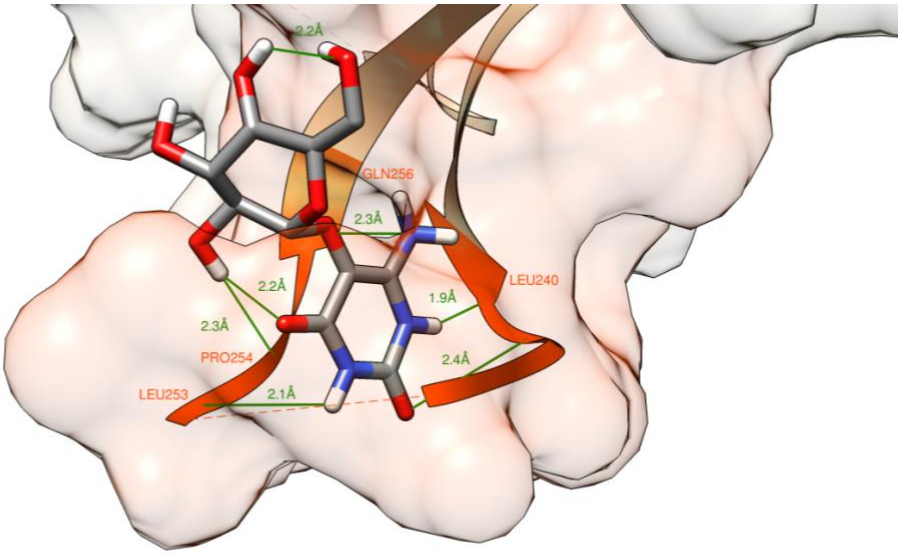

Figure 8. Binding mode analysis of convicine in the pocket 1 of AhR PAS-A domain.

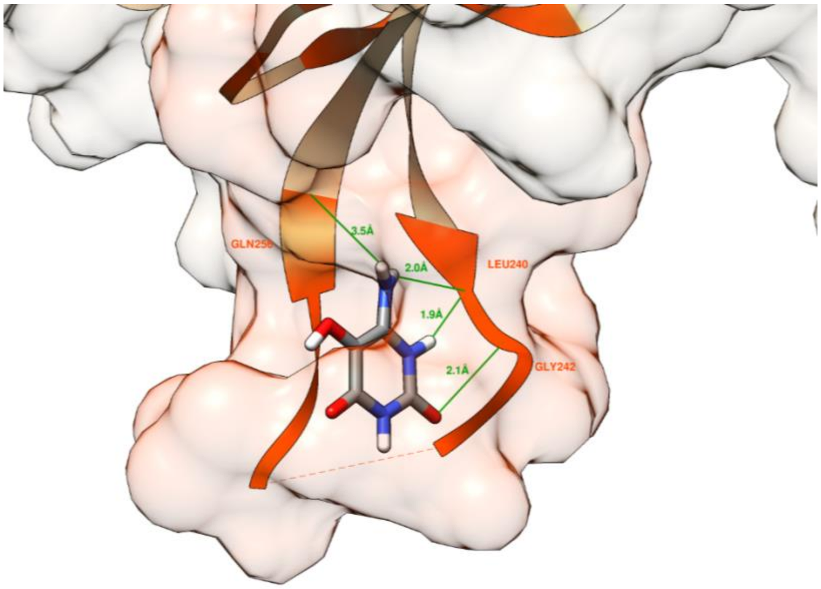

Figure 9. Binding mode analysis of isouramil (a metabolite derived from metabolism of convicine) in the pocket 1 of AhR PAS-A domain.

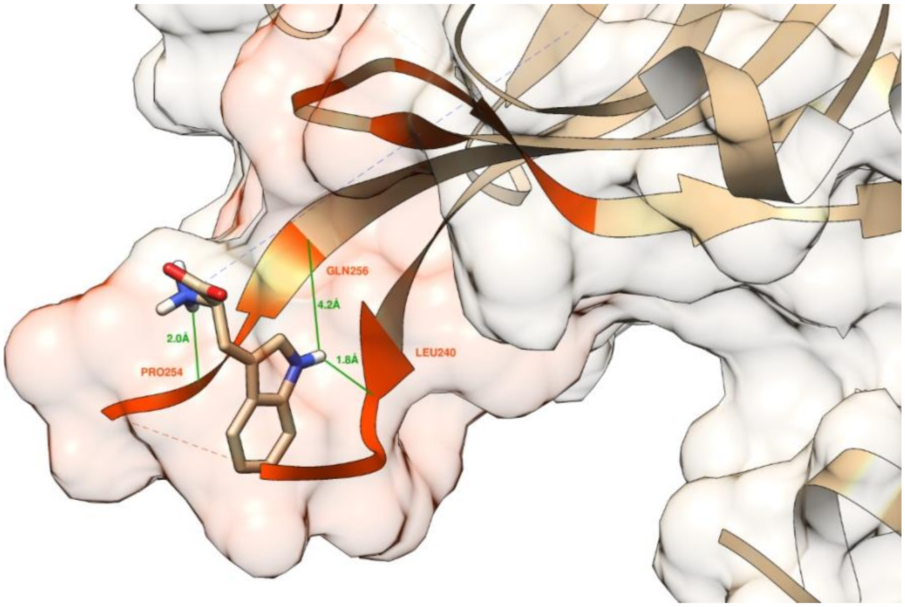

Figure 10. Binding site analysis of L-tryptophan in the pocket 1 of AhR PAS-A domain.

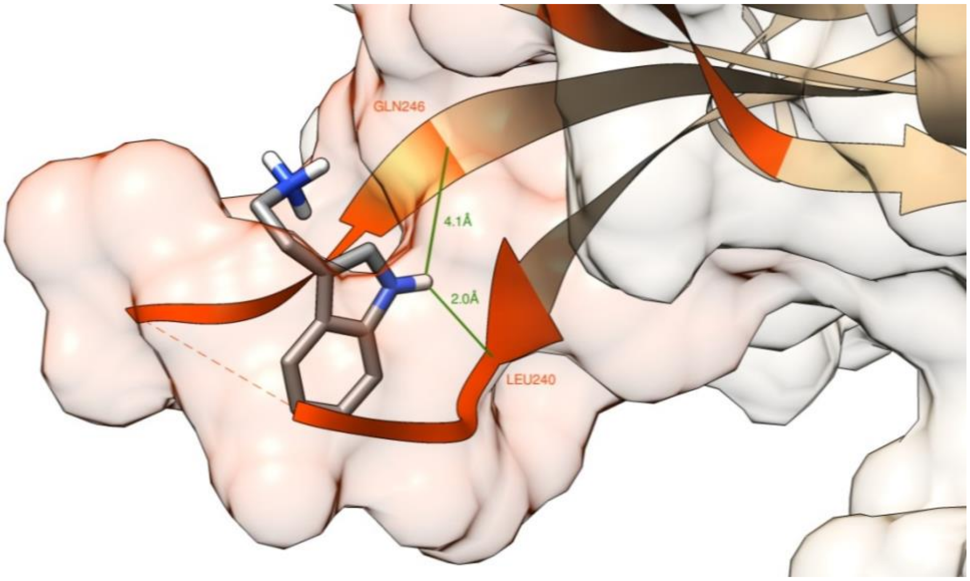

Figure 11. Binding site analysis of tryptamine (a metabolite derived from metabolism of L-tryptophan) in the pocket 1 of AhR PAS-A domain.

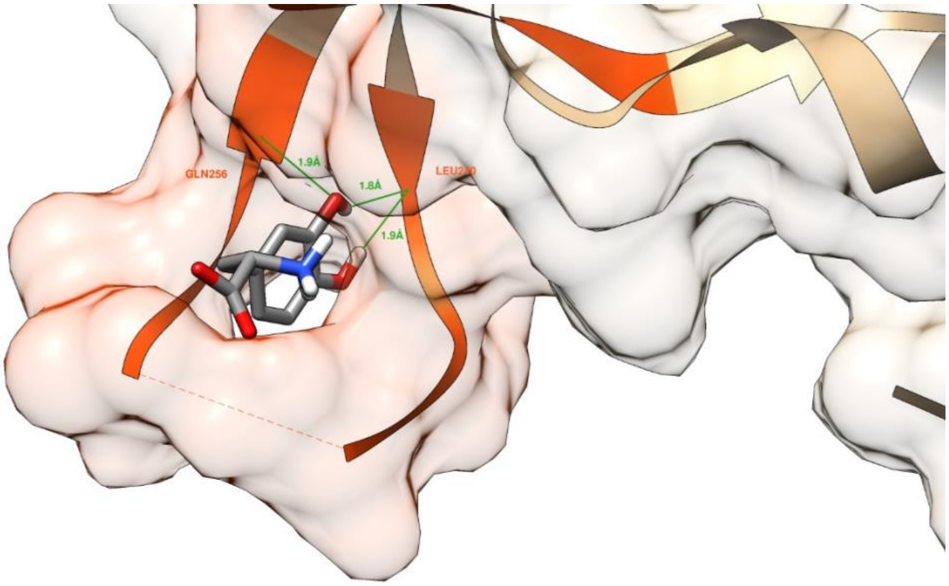

Figure 12. Binding mode analysis of L-DOPA in the pocket 1 of AhR PAS-A domain.

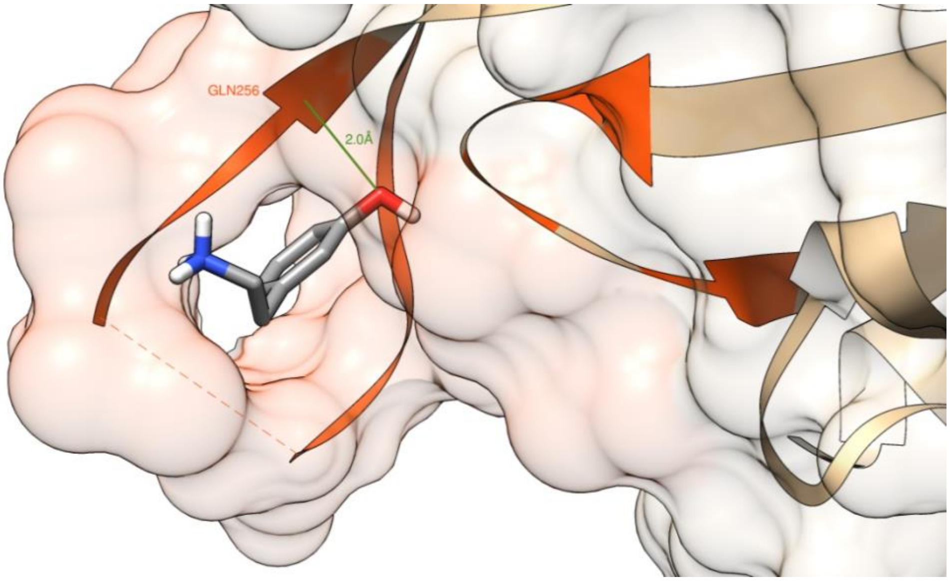

Figure 13. Binding mode analysis of m-Tyramine (a metabolite derived from metabolism of L-DOPA) in the pocket 1 of AhR PAS-A domain.

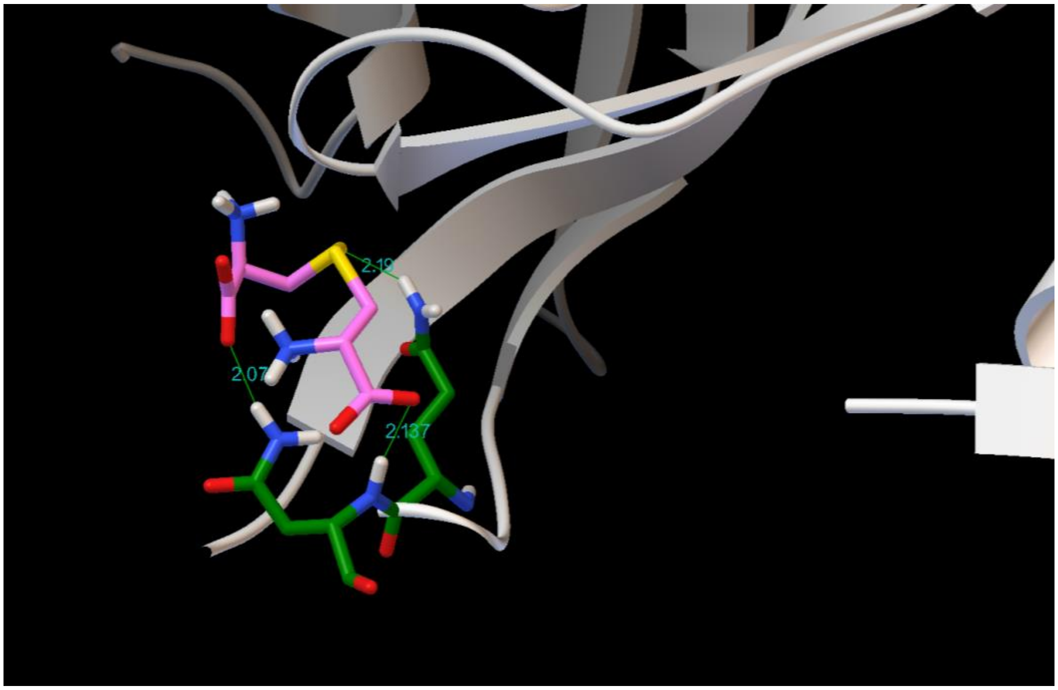

Figure 14. Binding mode analysis of L-cystine in the pocket 1 of AhR PAS-A domain. Modeling of L-Cystine was not possible in Chimera, but the presented interaction with AhR was obtained from AutoDock 4.2.

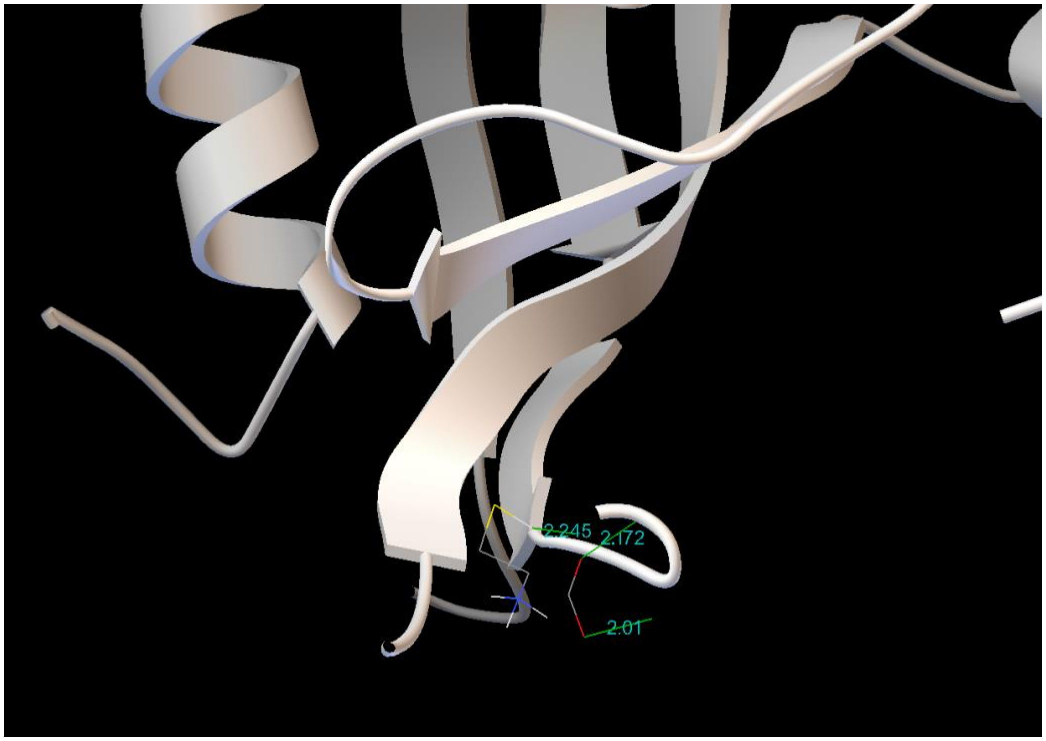

Figure 15. Binding mode analysis of L-cysteine (a metabolite derived from metabolism of L-cystine) in the pocket 1 of AhR PAS-A domain. Modeling of L-Cysteine was not possible in Chimera, but the presented interaction with AhR was obtained from AutoDock 4.2.

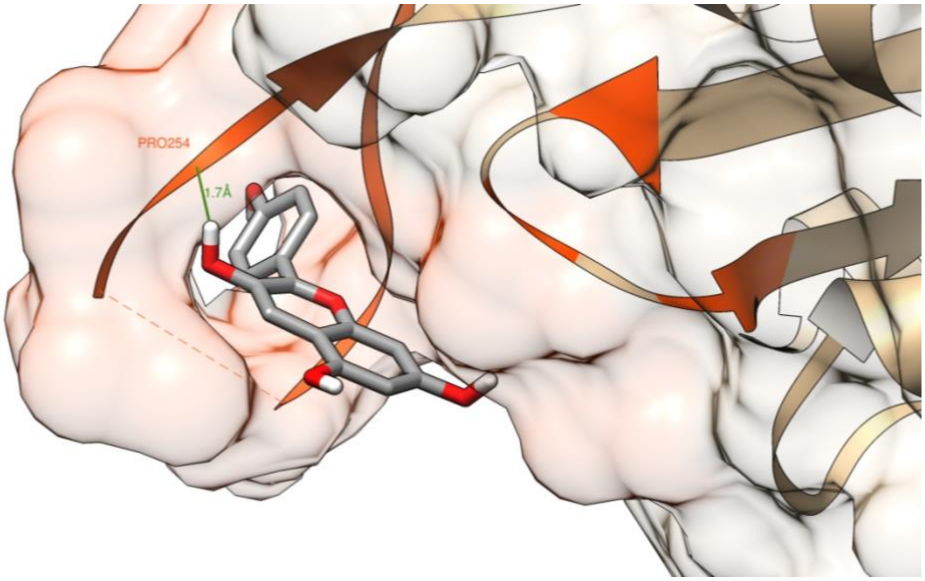

Figure 16. Binding mode analysis of pelargonidin in the pocket 1 of AhR PAS-A domain.

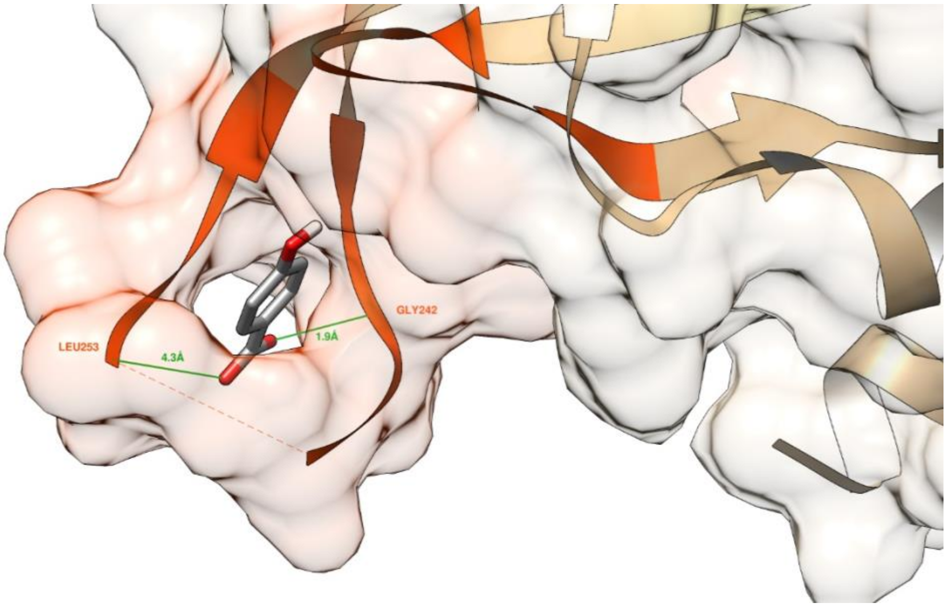

Figure 17. Binding mode analysis of 4-hydroxybenzoic acid (a metabolite derived from metabolism of pelargonidin) in the pocket 1 of AhR PAS-A domain.

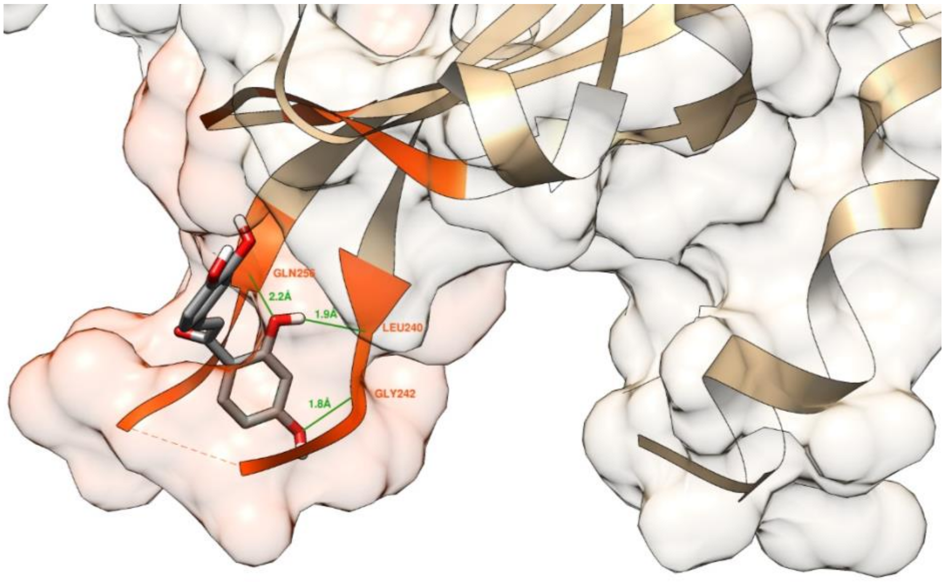

Figure 18. Binding mode analysis of butein in the pocket 1 of AhR PAS-A domain.

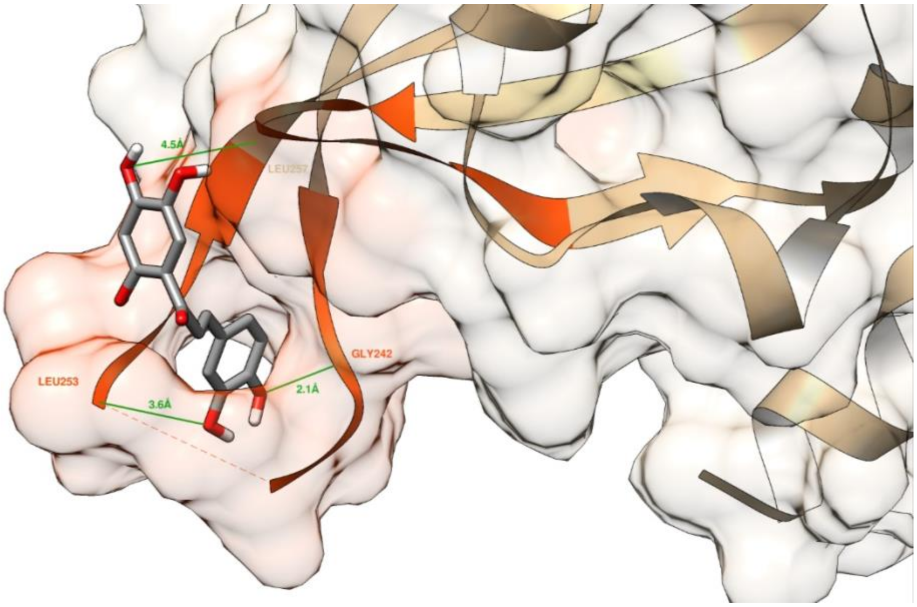

Figure 19. Binding mode analysis of Neoplathymenin (a metabolite derived from metabolism of butein) in the pocket 1 of AhR PAS-A domain.

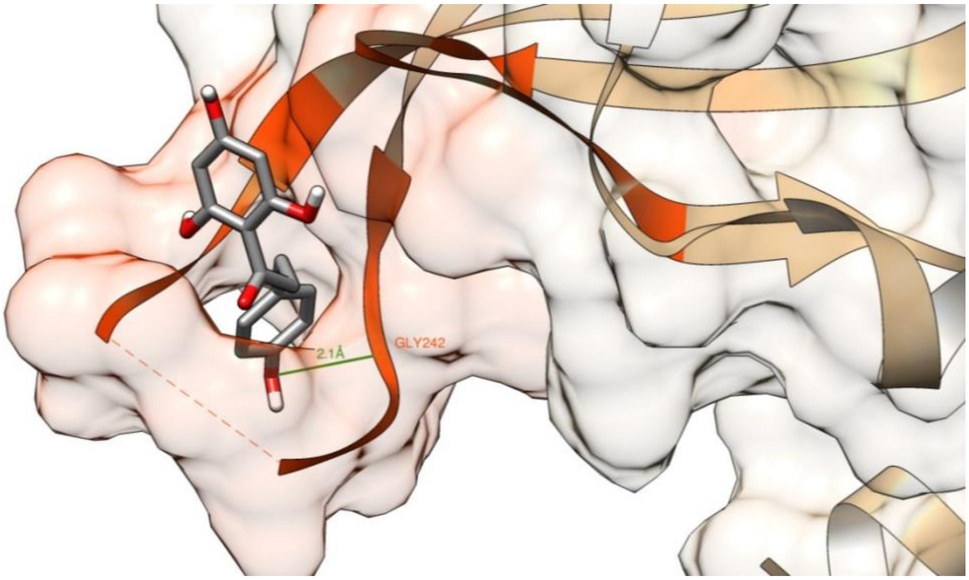

Figure 20. Binding mode analysis of phloretin in the pocket 1 of AhR PAS-A domain.

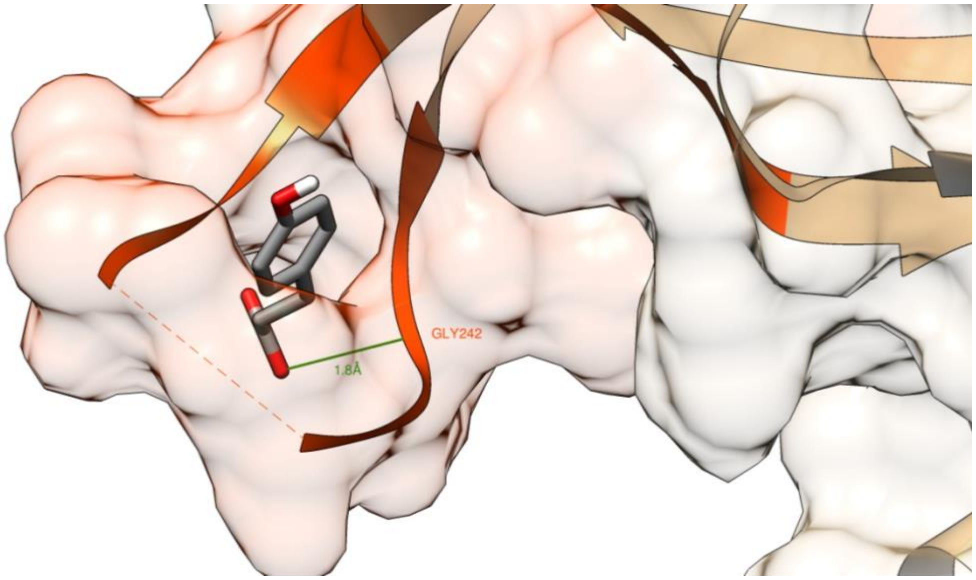

Figure 21. Binding mode analysis of phloretic acid (a metabolite derived from metabolism of phloretin) in the pocket 1 of AhR PAS-A domain.

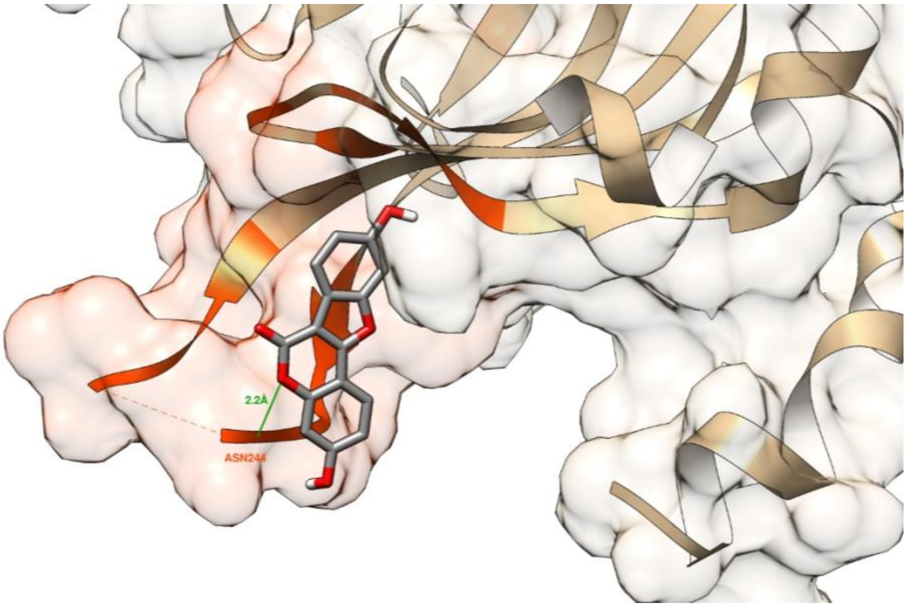

Figure 22. Binding mode analysis of coumestrol in the pocket 1 of AhR PAS-A domain.

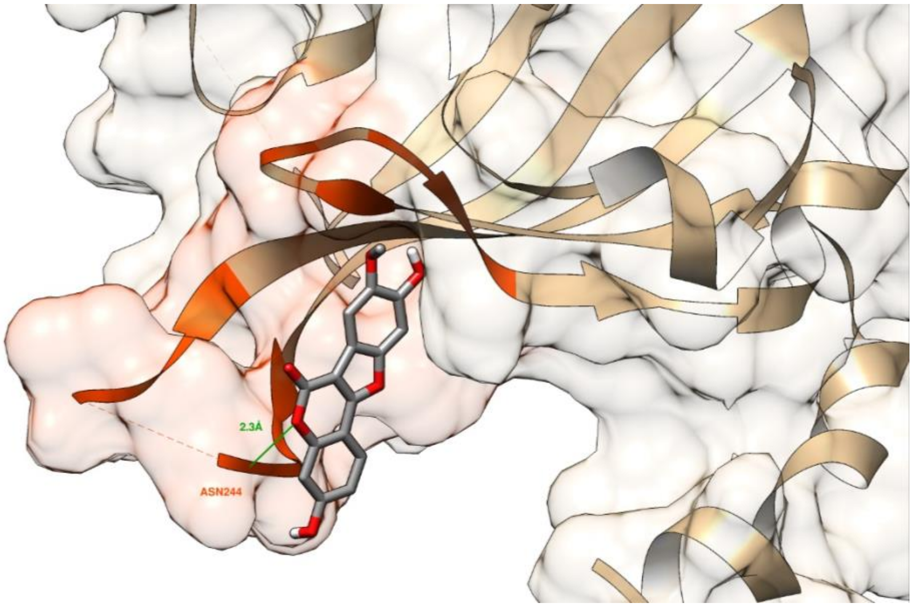

Figure 23. Binding mode analysis of 8-methoxycoumestrol (a metabolite derived from metabolism of coumestrol) in the pocket 1 of AhR PAS-A domain.

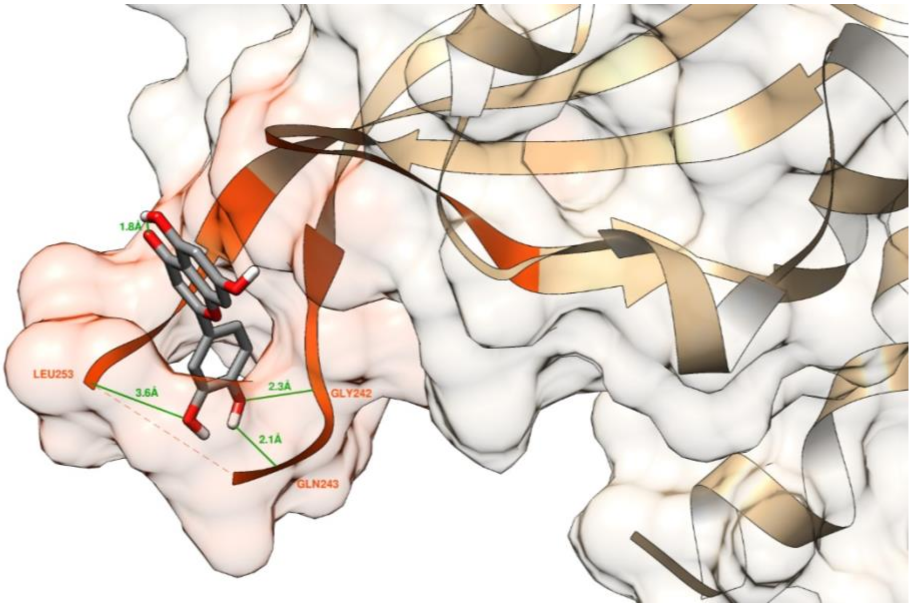

Figure 24. Binding mode analysis of luteolin in the pocket 1 of AhR PAS-A domain.

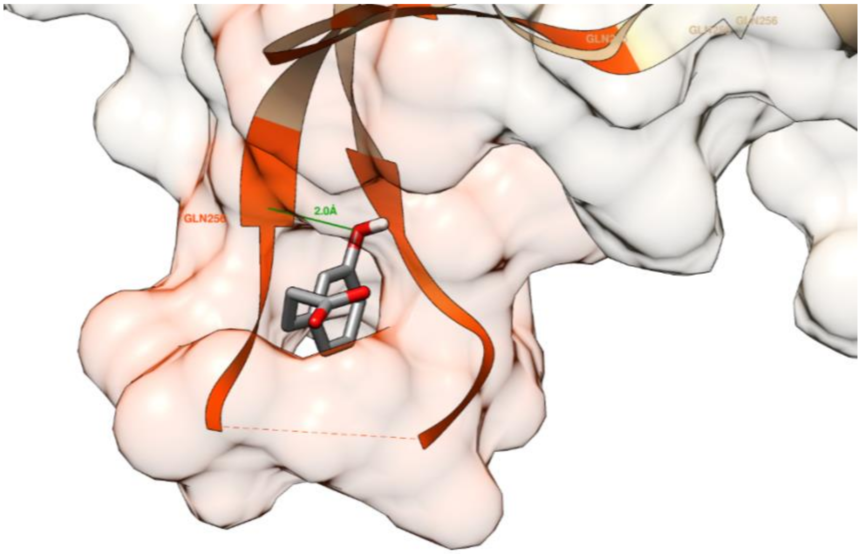

Figure 25. Binding mode analysis of 3-(3-hydroxyphenyl)-propionic acid (a metabolite derived from metabolism of luteolin) in the pocket 1 of AhR PAS-A domain.

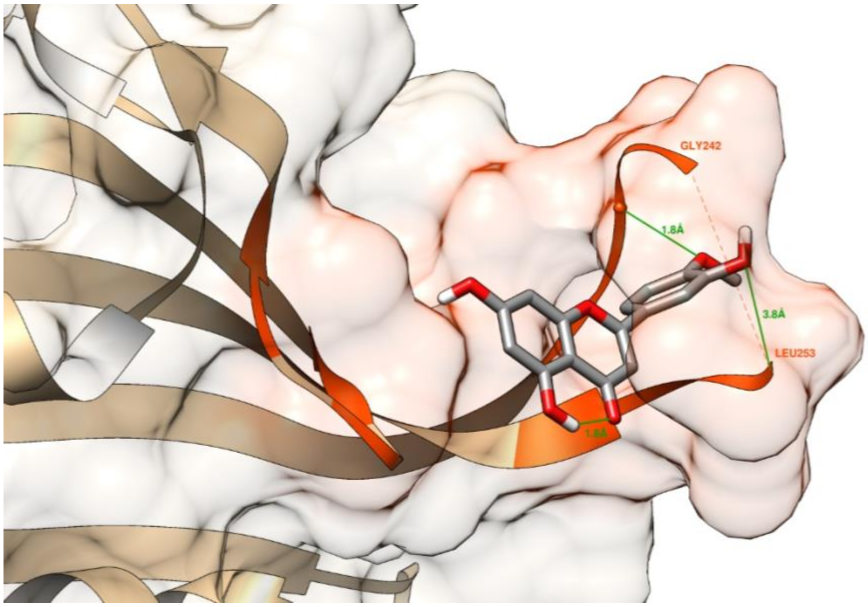

Figure 26. Binding mode analysis of diosmetin in the pocket 1 of AhR PAS-A domain.

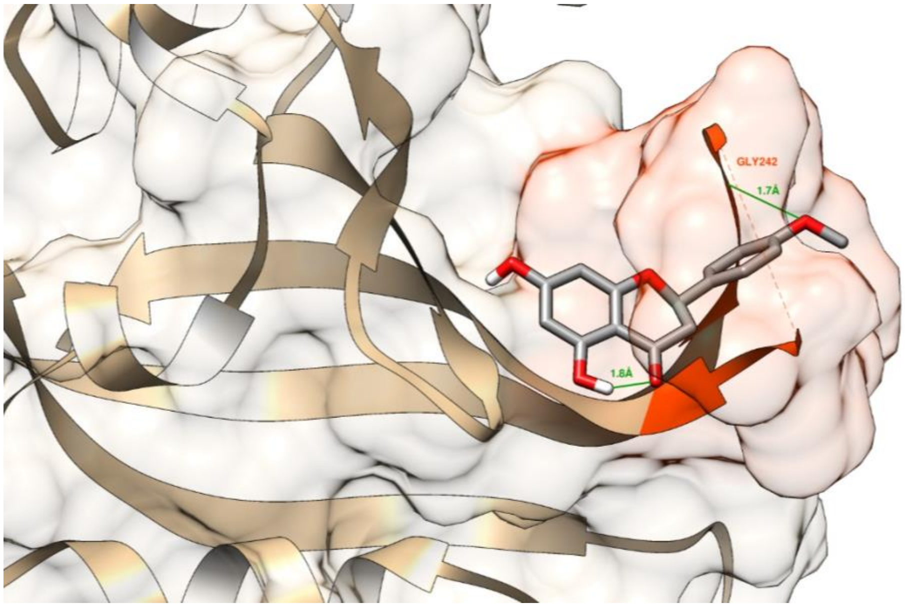

Figure 27. Binding mode analysis of citrifoliol (a metabolite derived from metabolism of diosmetin) in the pocket 1 of AhR PAS-A domain.

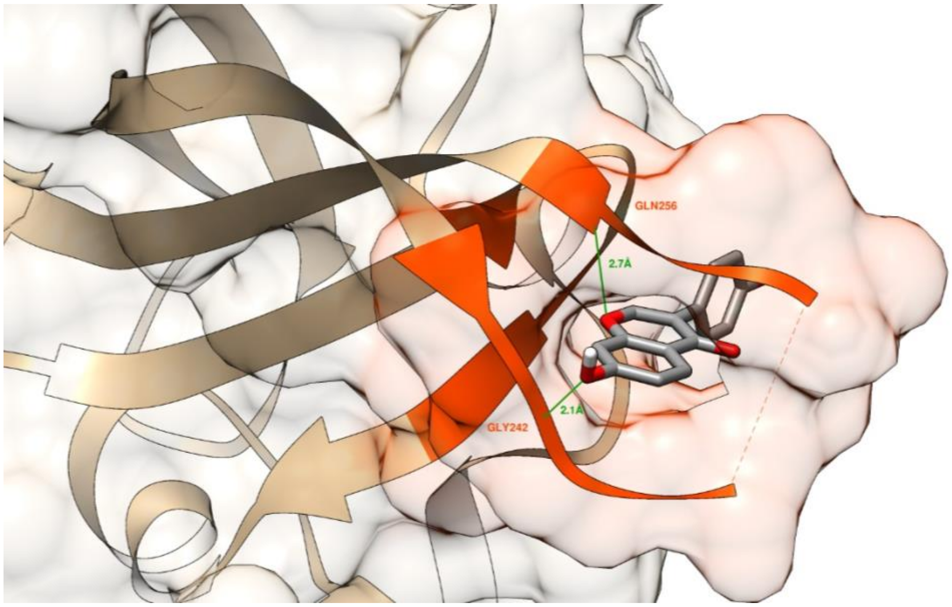

Figure 28. Binding mode analysis of daidzein in the pocket 1 of AhR PAS-A domain.

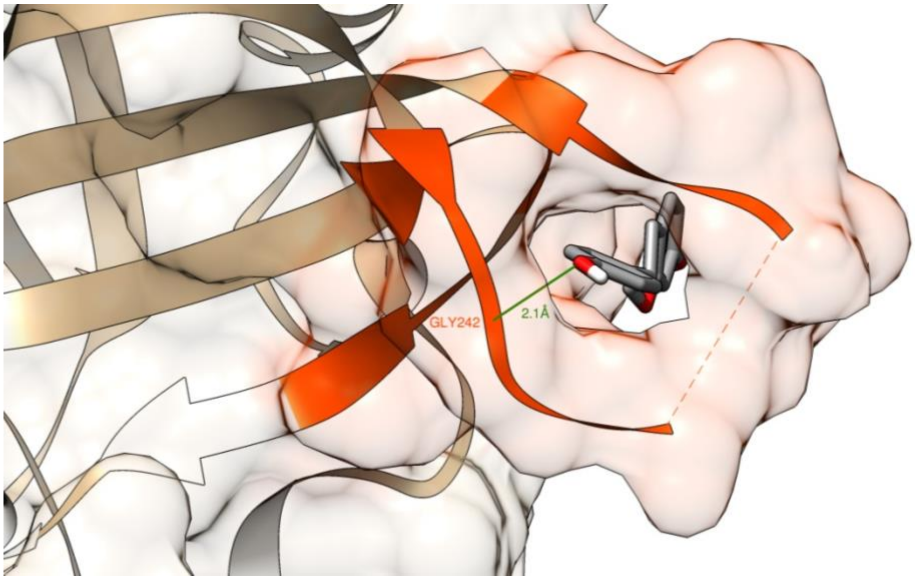

Figure 29. Binding mode analysis of S-equol (a metabolite derived from metabolism of daidzein) in the pocket 1 of AhR PAS-A domain.

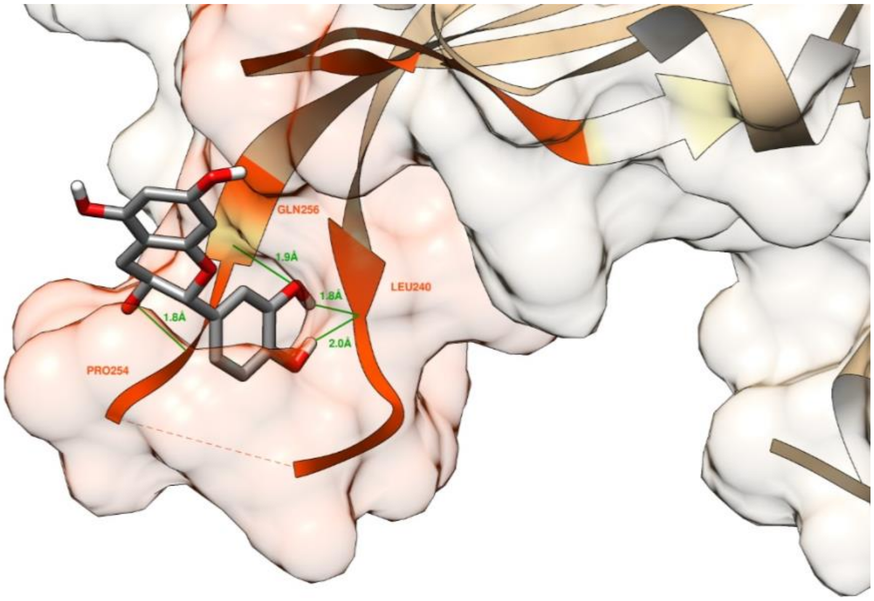

Figure 30. Binding mode analysis of catechin in the pocket 1 of AhR PAS-A domain.

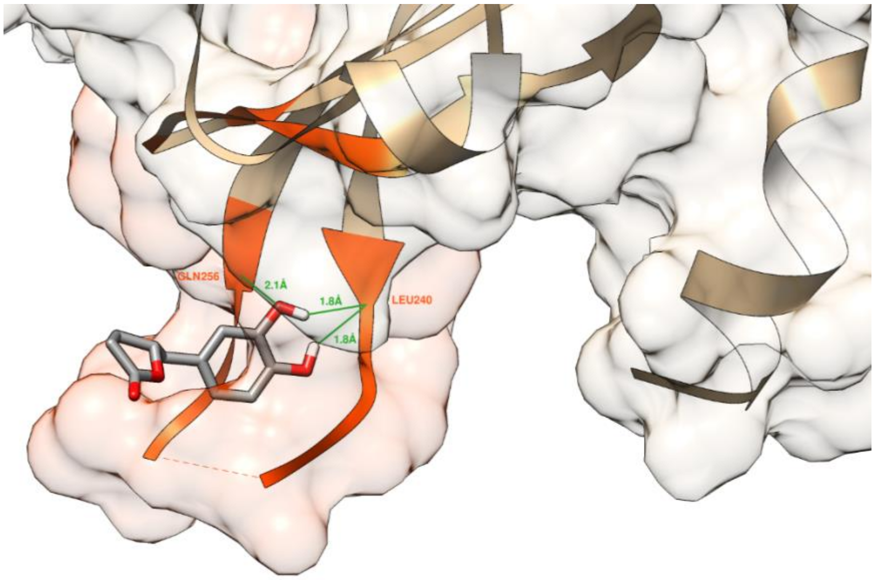

Figure 31. Binding mode analysis of 5-(3',4'-dihydroxyphenyl)-gamma-valerolactone (a metabolite derived from metabolism of catechin) in the pocket 1 of AhR PAS-A domain.

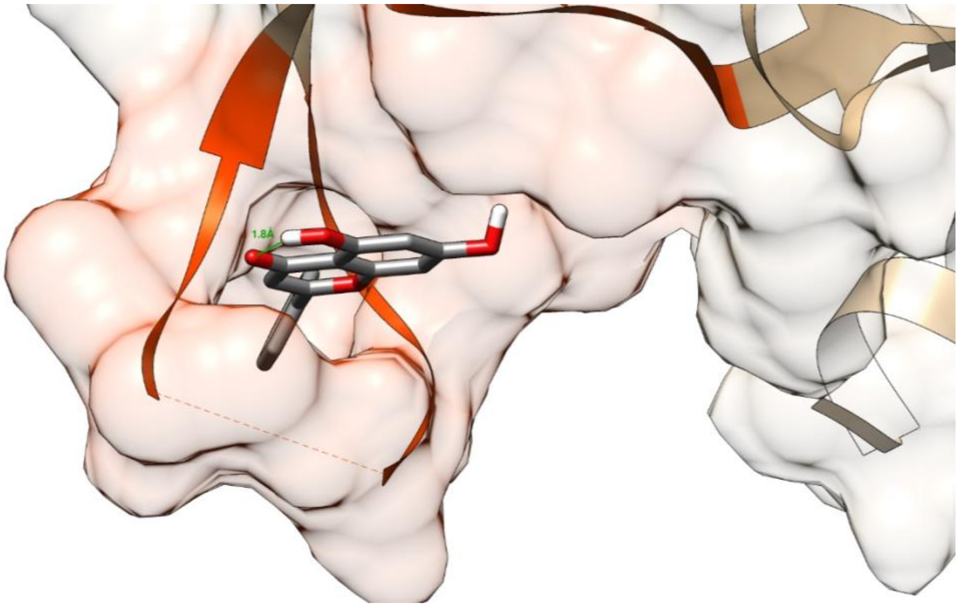

Figure 32. Binding mode analysis of chrysin in the pocket 1 of AhR PAS-A domain.

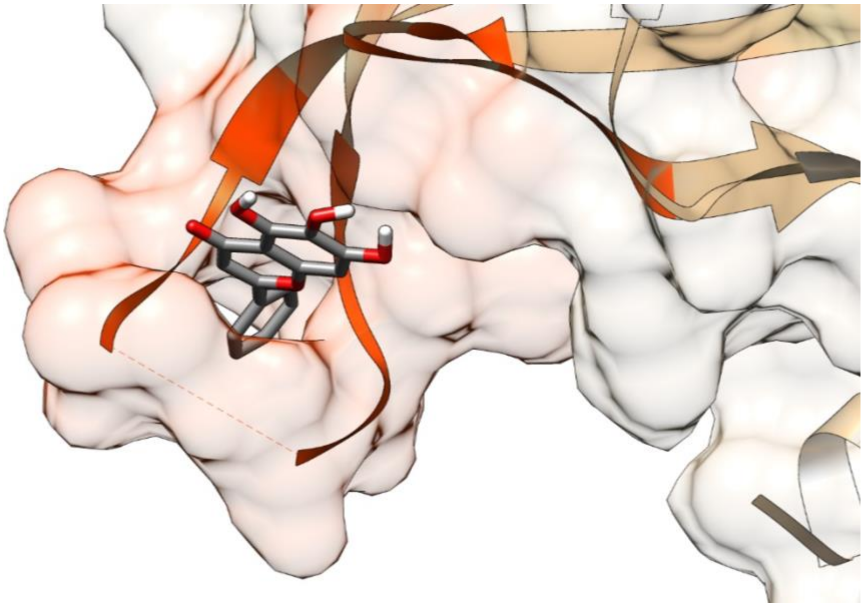

Figure 33. Binding mode analysis of baicalein (a metabolite derived from metabolism of chrysin) in the pocket 1 of AhR PAS-A domain.

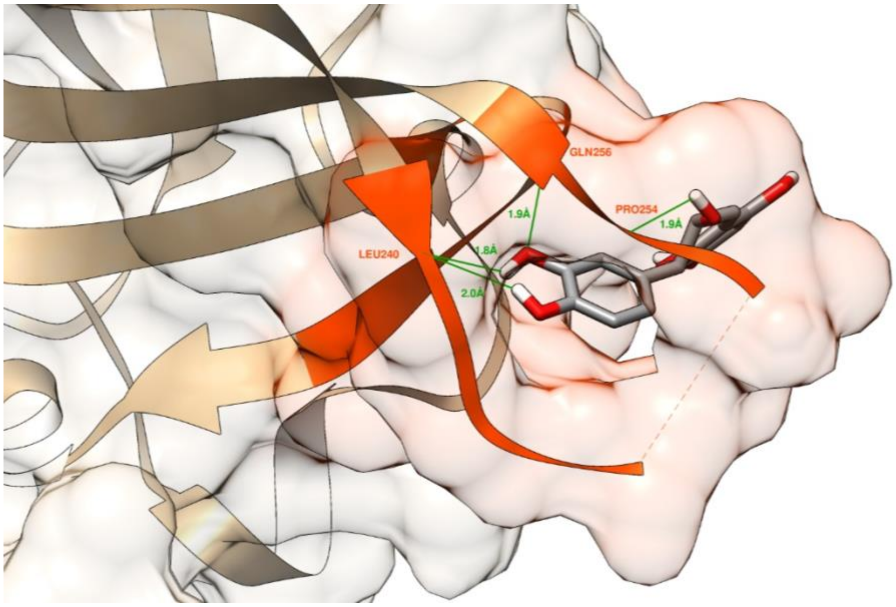

Figure 34. Binding mode analysis of epicatechin in the pocket 1 of AhR PAS-A domain.

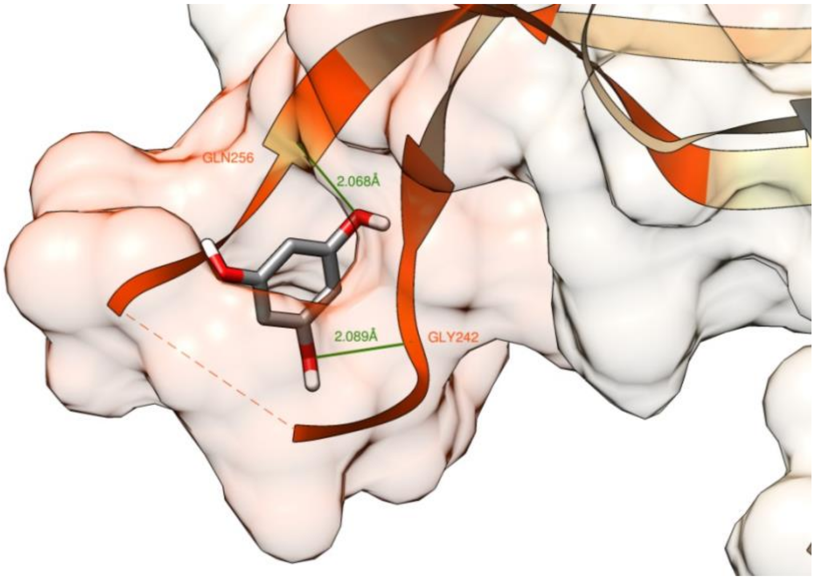

Figure 35. Binding mode analysis of Phloroglucinol (a metabolite derived from metabolism of epicatechin) in the pocket 1 of AhR PAS-A domain.

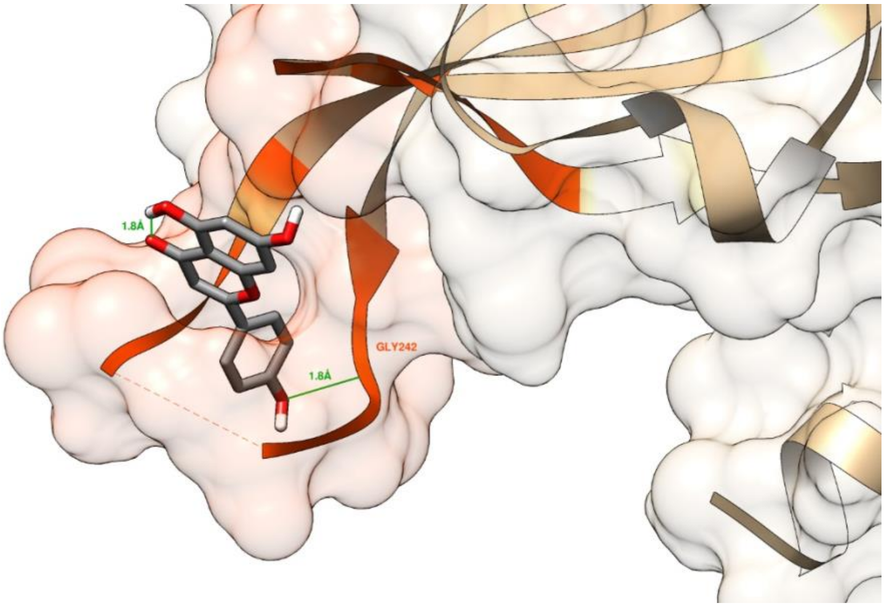

Figure 36. Binding mode analysis of apigenin in the pocket 1 of AhR PAS-A domain.

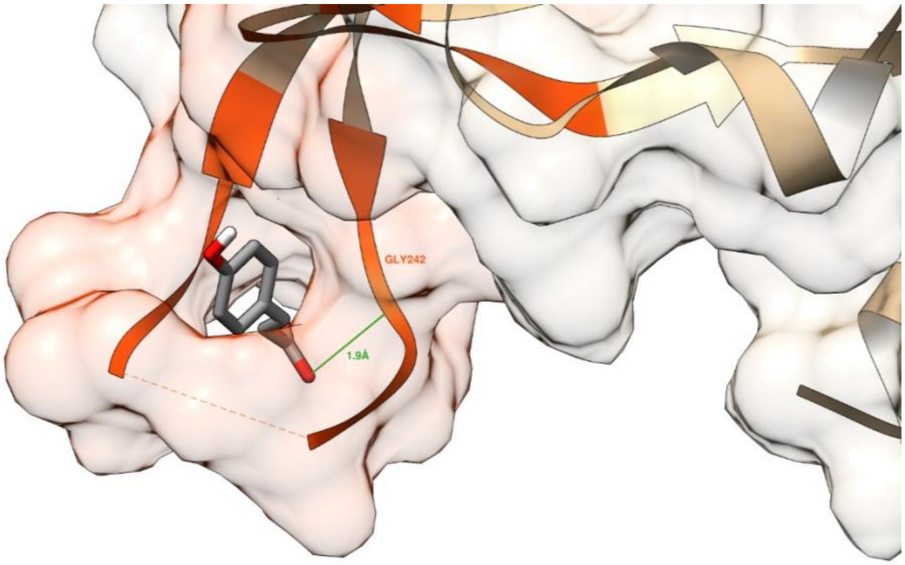

Figure 37. Binding mode analysis of 4-hydroxycinnamic acid (a metabolite derived from metabolism of apigenin) in the pocket 1 of AhR PAS-A domain.

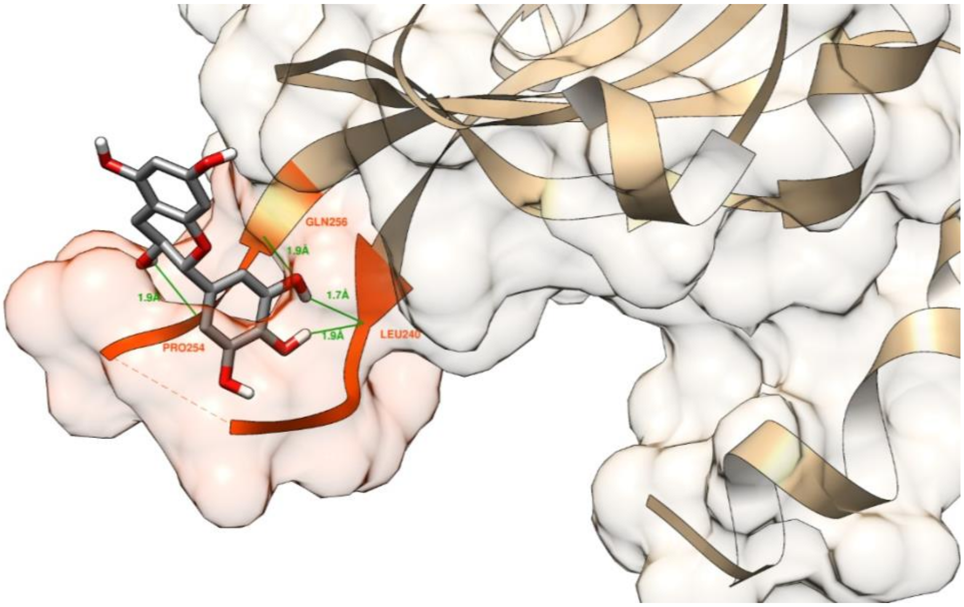

Figure 38. Binding mode analysis of gallocatechin in the pocket 1 of AhR PAS-A domain.

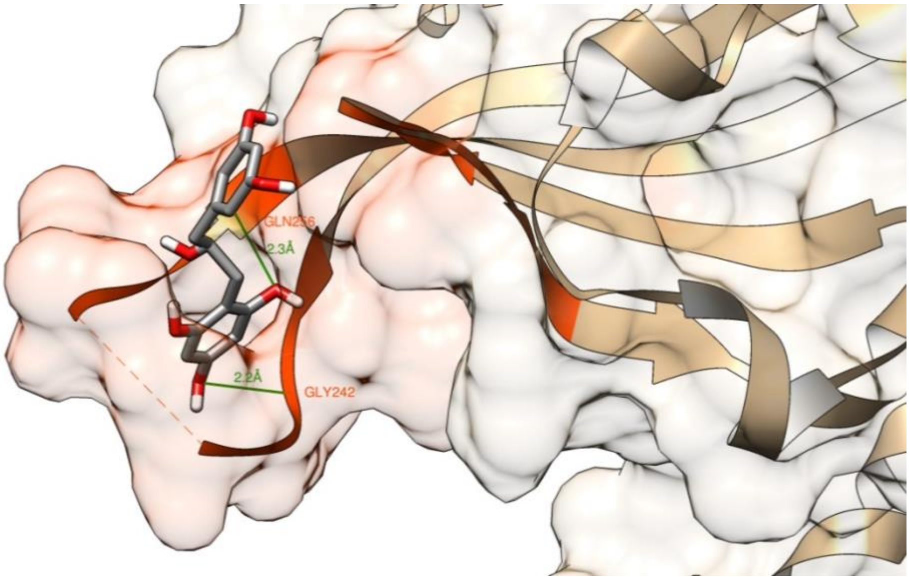

Figure 39. Binding mode analysis of 1-(3,4,5-trihydroxyphenyl)-3-(2,4,6-trihydroxyphenyl)-2-propanol (a metabolite derived from metabolism of gallocatechin) in the pocket 1 of AhR PAS-A domain.

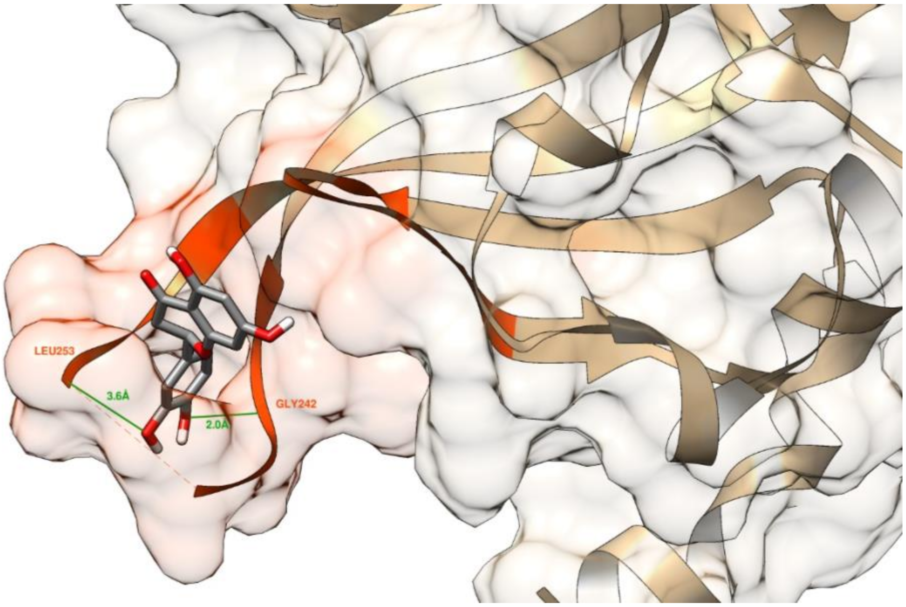

Figure 40. Binding mode analysis of eriodyctiol in the pocket 1 of AhR PAS-A domain.

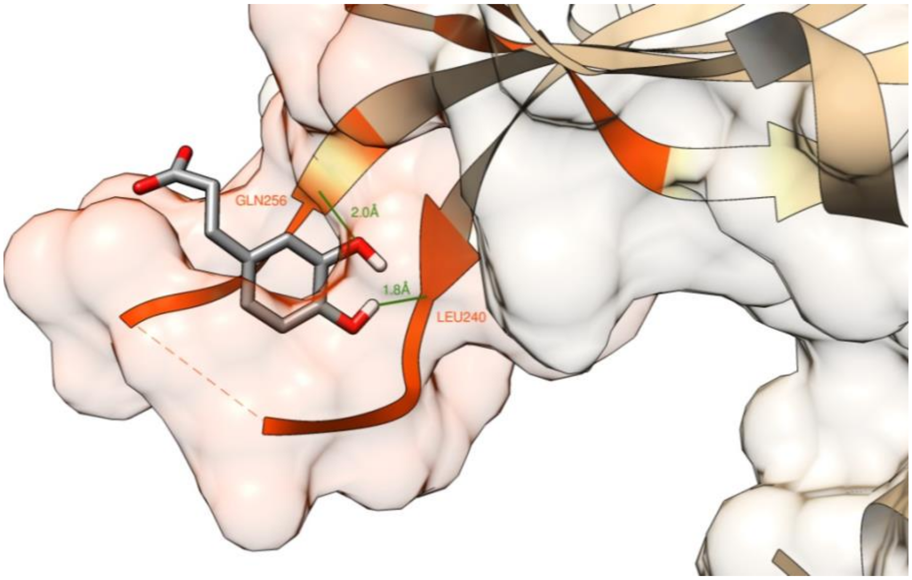

Figure 41. Binding mode analysis of 3-(3,4-dihydroxyphenyl)propionic acid (a metabolite derived from metabolism of eriodyctiol) in the pocket 1 of AhR PAS-A domain.

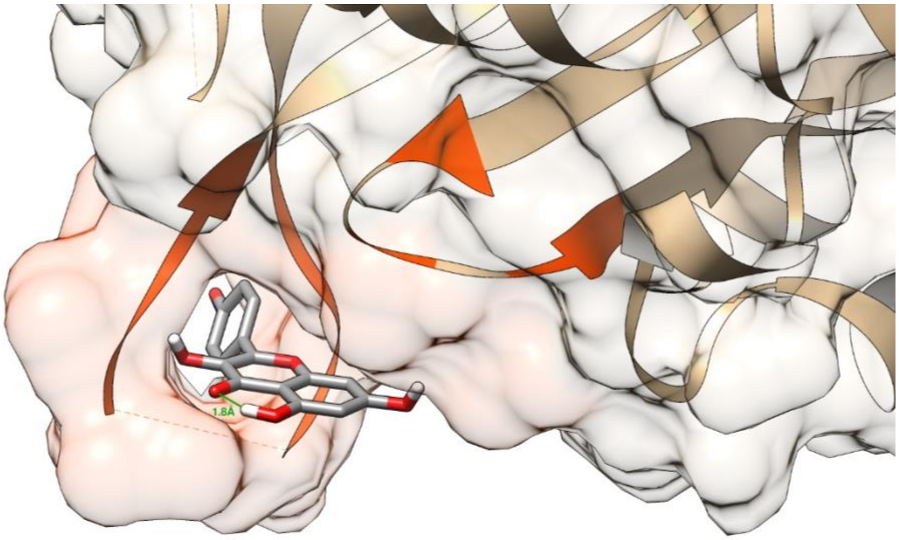

Figure 42. Binding mode analysis of kaempferol in the pocket 1 of AhR PAS-A domain.

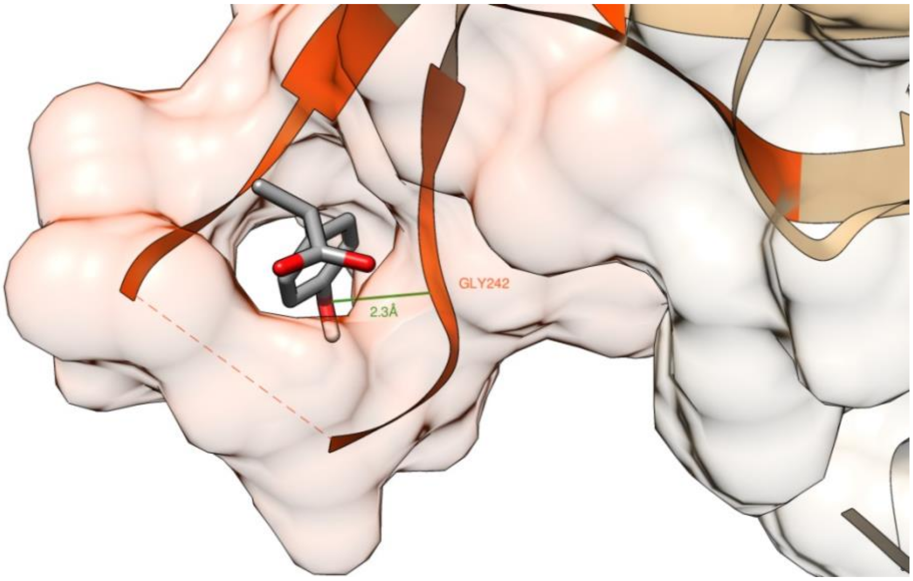

Figure 43. Binding mode analysis of 2-(4-hydroxyphenyl)propionic acid (a metabolite derived from metabolism of kaempferol) in the pocket 1 of AhR PAS-A domain.

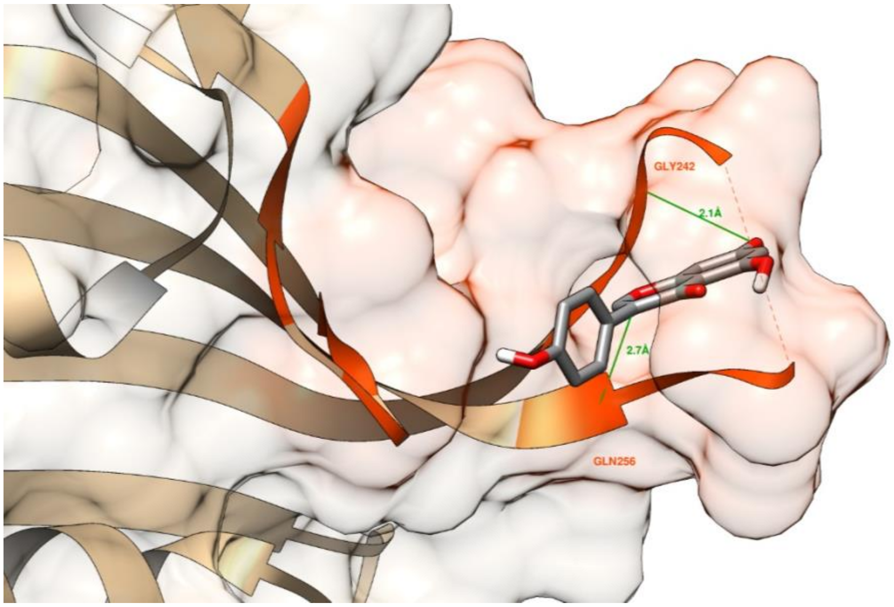

Figure 44. Binding mode analysis of genistein in the pocket 1 of AhR PAS-A domain.

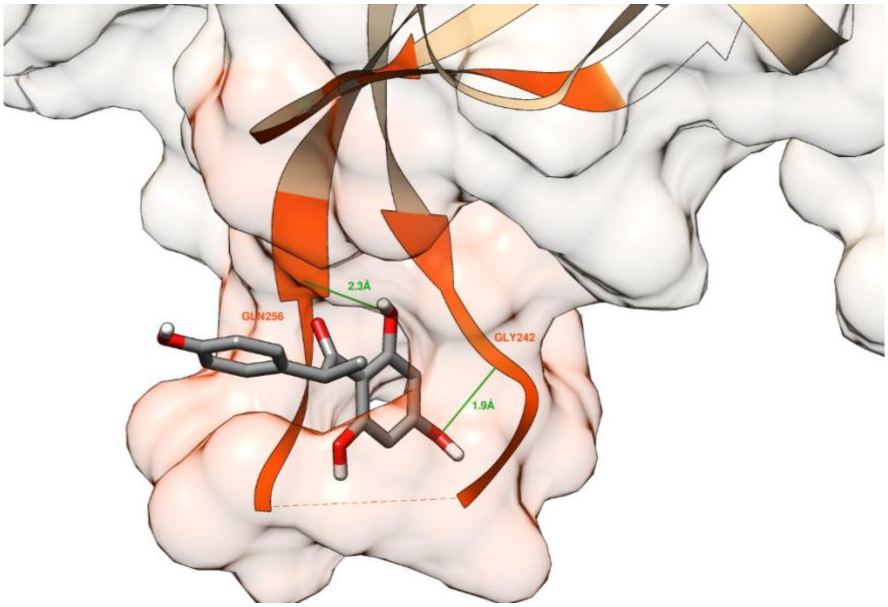

Figure 45. Binding site analysis of 6'-hydroxy-O-desmethylangolensin (a metabolite derived from metabolism of genistein) in the pocket 1 of AhR PAS-A domain.

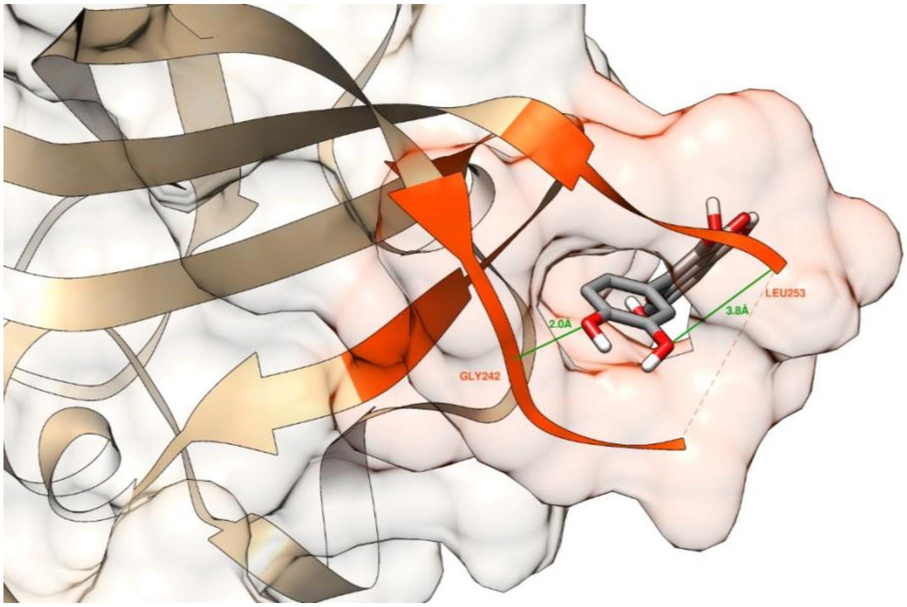

Figure 46. Binding mode analysis of quercetin in the pocket 1 of AhR PAS-A domain.

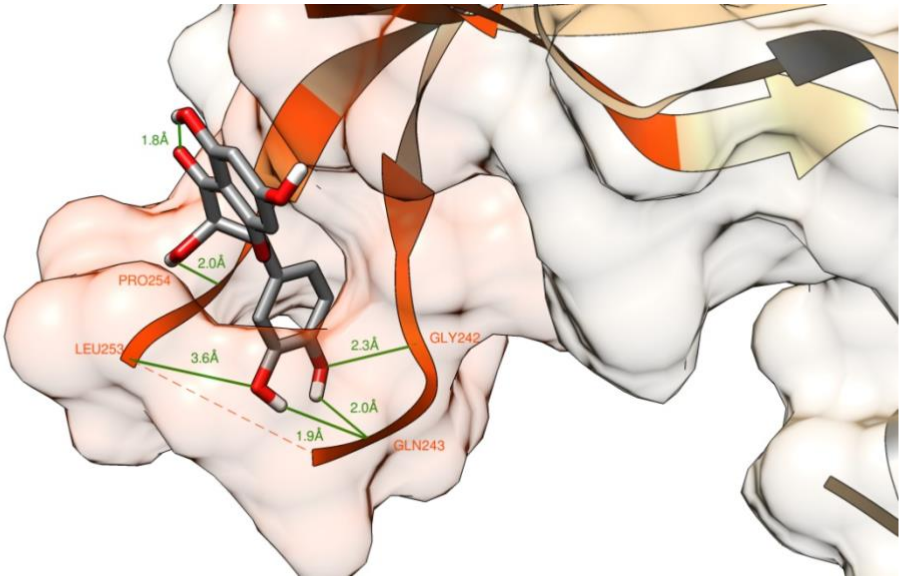

Figure 47. Binding mode analysis of taxifolin (a metabolite derived from metabolism of quercetin) in the pocket 1 of AhR PAS-A domain.

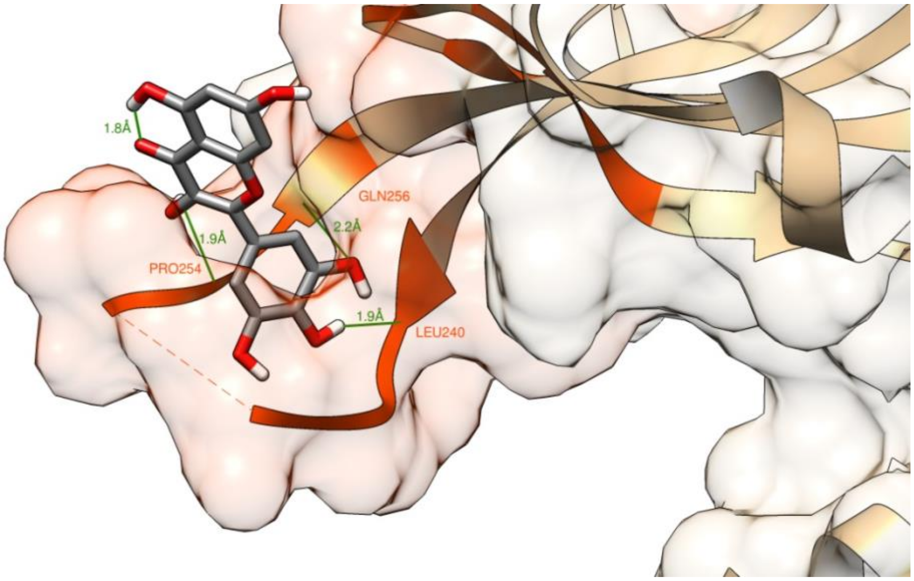

Figure 48. Binding mode analysis of myricetin in the pocket 1 of AhR PAS-A domain.

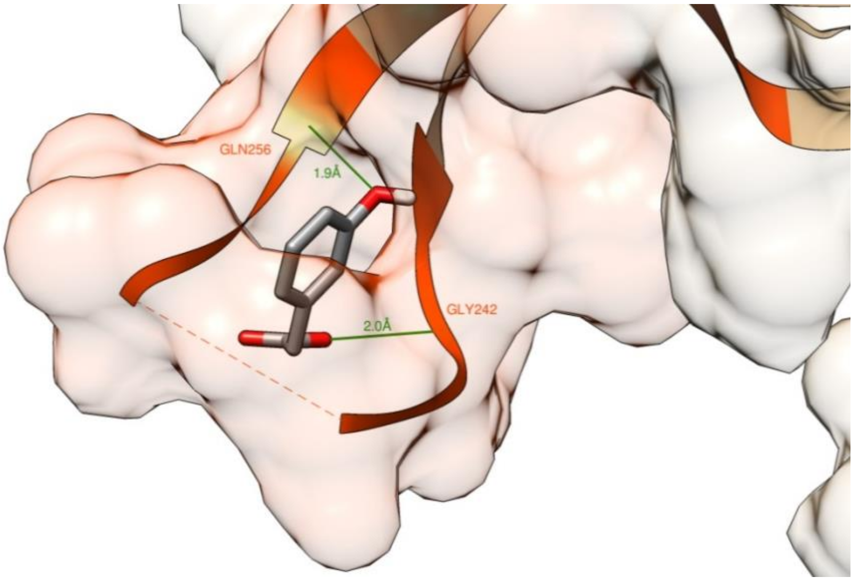

Figure 49. Binding mode analysis of 2-(3-hydroxyphenyl)acetic acid (a metabolite derived from metabolism of myricetin) in the pocket 1 of AhR PAS-A domain.

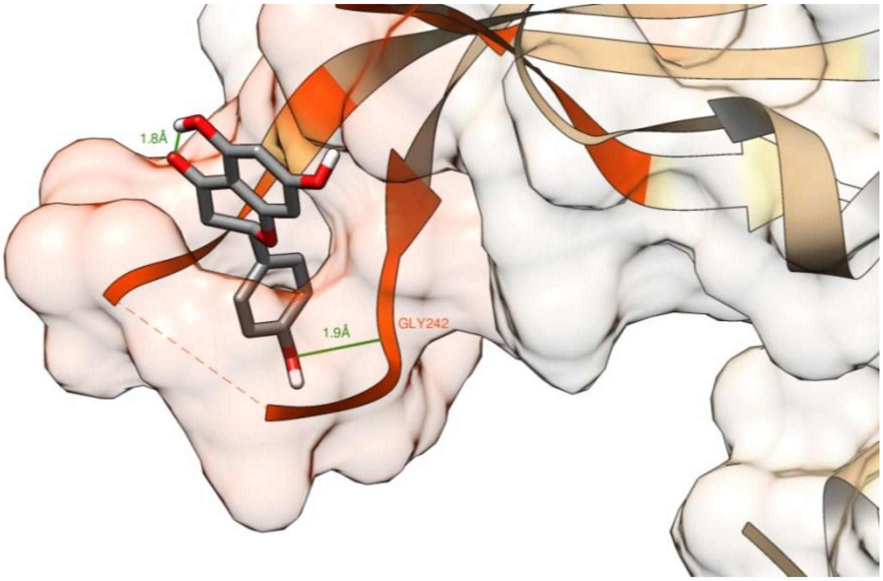

Figure 50. Binding mode analysis of naringenin in the pocket 1 of AhR PAS-A domain.

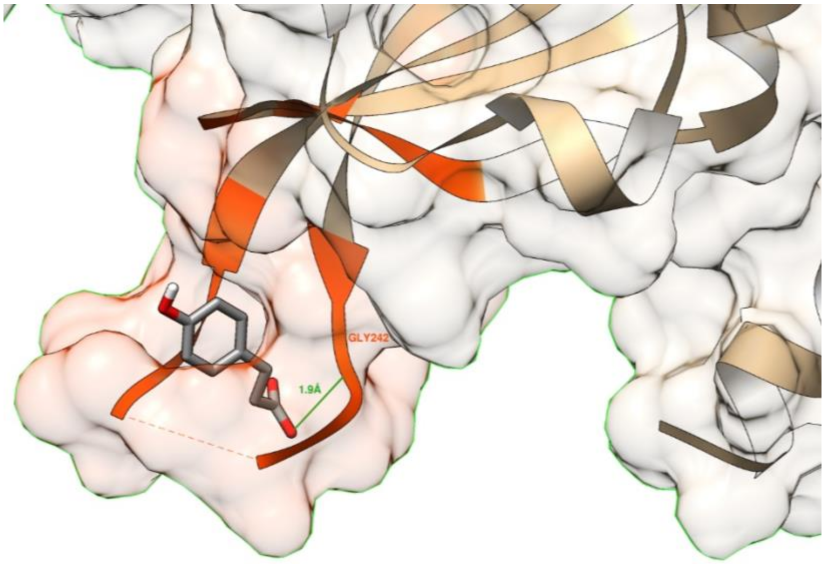

Figure 51. Binding mode analysis of 3-(4-hydroxyphenyl)propionic acid (a metabolite derived from metabolism of naringenin) in the pocket 1 of AhR PAS-A domain.

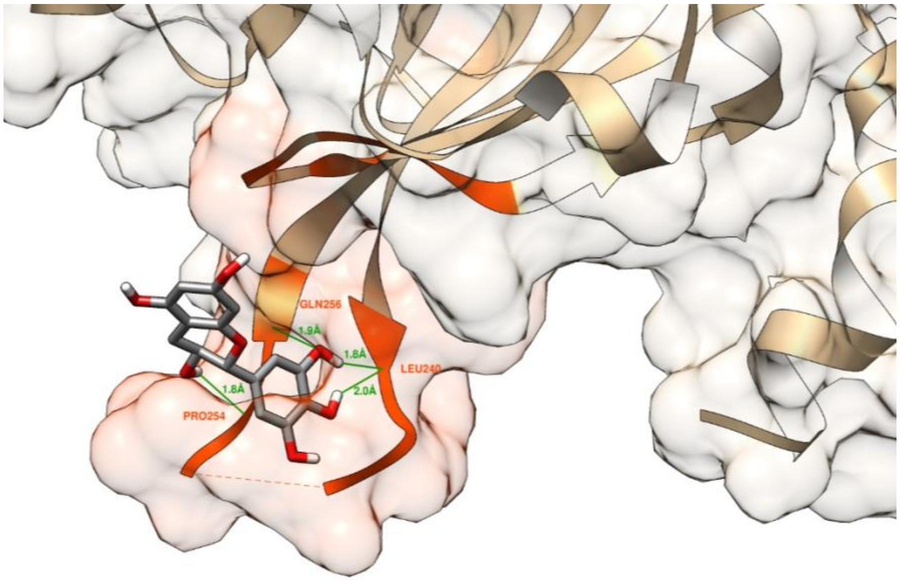

Figure 52. Binding mode analysis of epigallocatechin in the pocket 1 of AhR PAS-A domain.

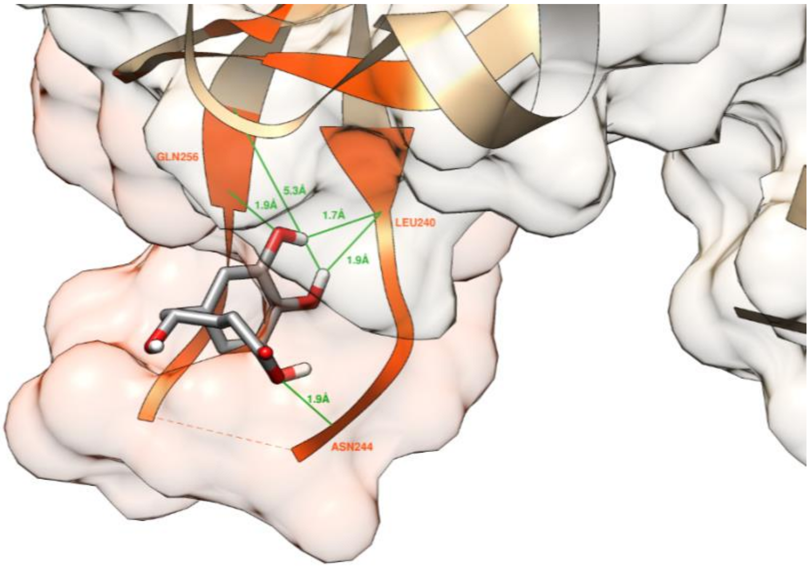

Figure 53. Binding mode analysis of 4-hydroxy-5-(3,4,5-trihydroxyphenyl)valeric acid (a metabolite derived from epigallocatechin) in the pocket 1 of AhR PAS-A domain.

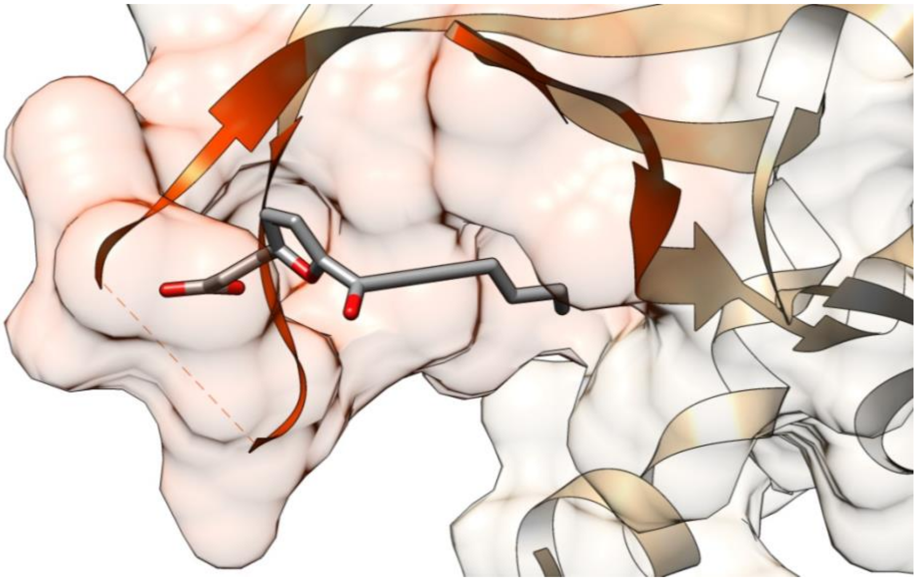

Figure 54. Binding mode analysis of wyerone acid in the pocket 1 of AhR PAS-A domain.

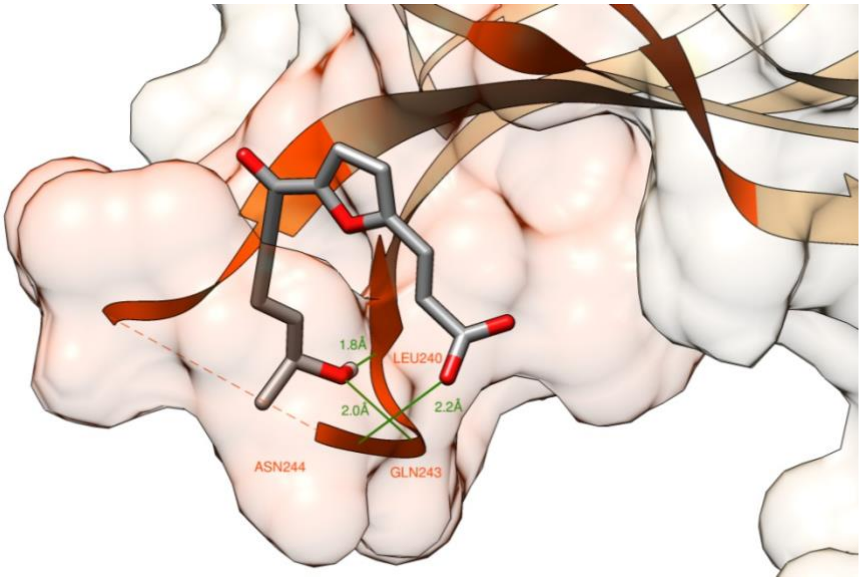

Figure 55. Binding mode analysis of (E)-3-(5-((E)-6-hydroxyhept-4-en-2-ynoyl)furan-2-yl)acrylic acid (a metabolite derived from metabolism of wyerone acid) in the pocket 1 of AhR PAS-A domain.

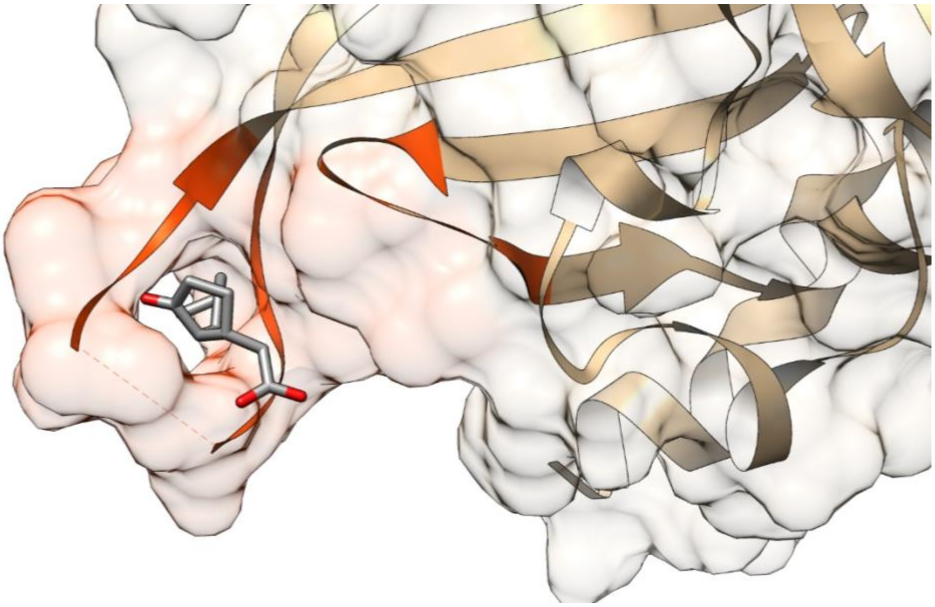

Figure 56. Binding mode analysis of jasmonic acid in the pocket 1 of AhR PAS-A domain.

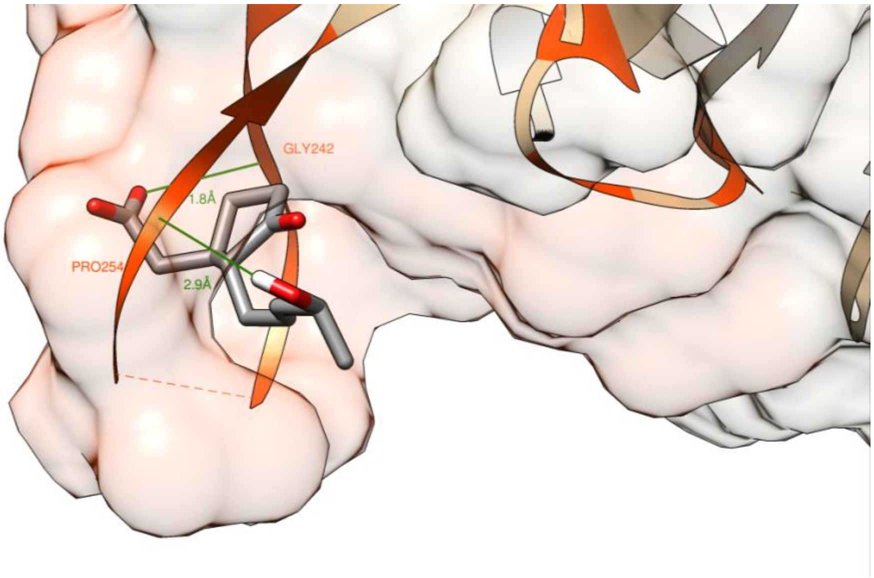

Figure 57. Binding mode analysis of 2-((1R,2R)-2-((E)-4-hydroxypent-2-en-1-yl)-3-oxocyclopentyl)acetic acid (a metabolite derived from metabolism of jasmonic acid) in the pocket 1 of AhR PAS-A domain.

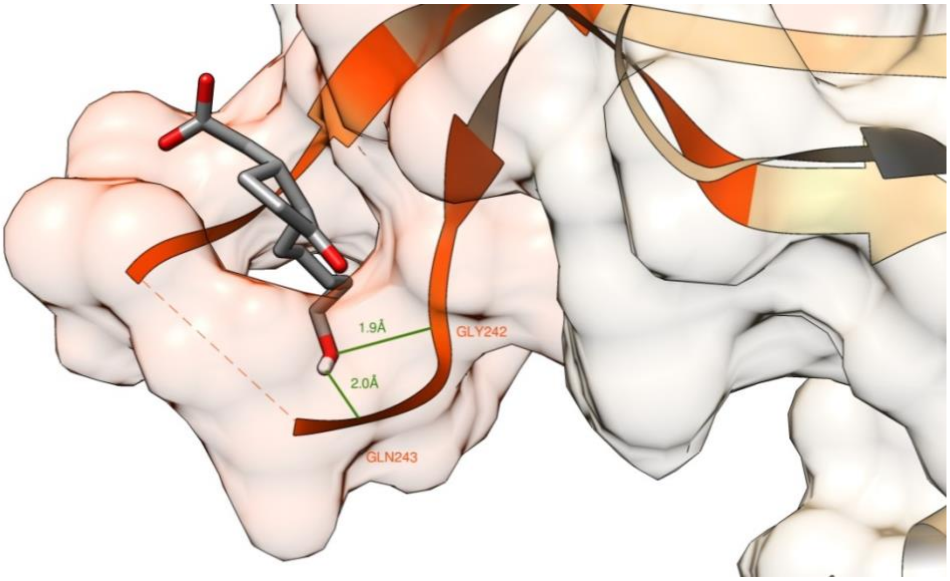

Figure 58. Binding mode analysis of tuberonic acid in the pocket 1 of AhR PAS-A domain.

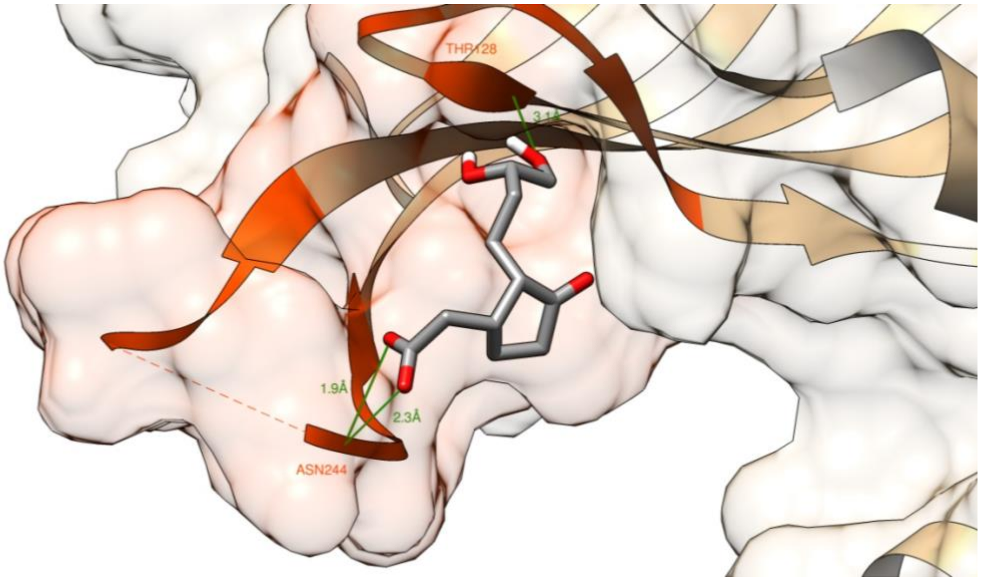

Figure 59. Binding mode analysis of 2-((1R,2S)-2-(3,4-dihydroxybutyl)-3-oxocyclopentyl)acetic acid (a metabolite derived from metabolism of tuberonic acid) in the pocket 1 of AhR PAS-A domain.

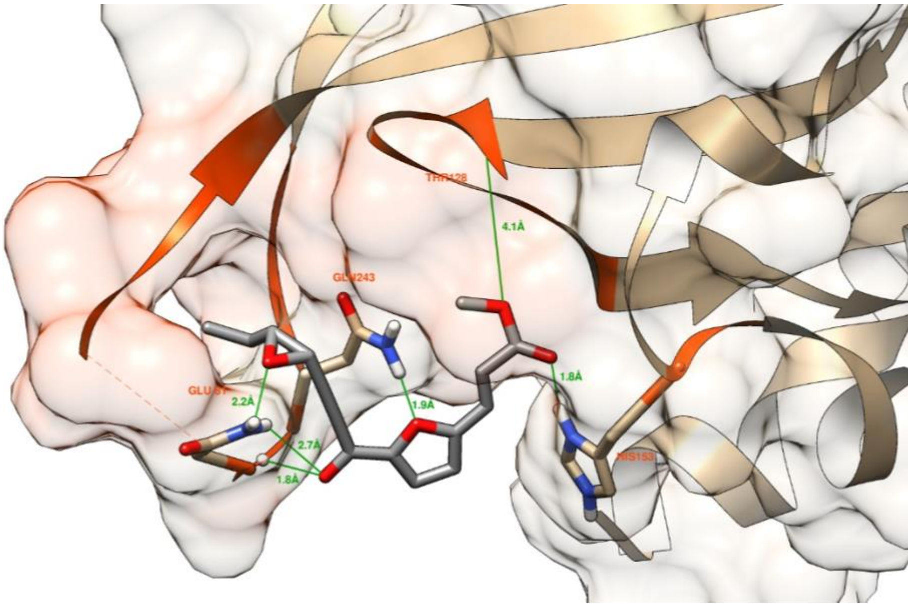

Figure 60. Binding mode analysis of wyerone epoxide in the pocket 1 of AhR PAS-A domain.

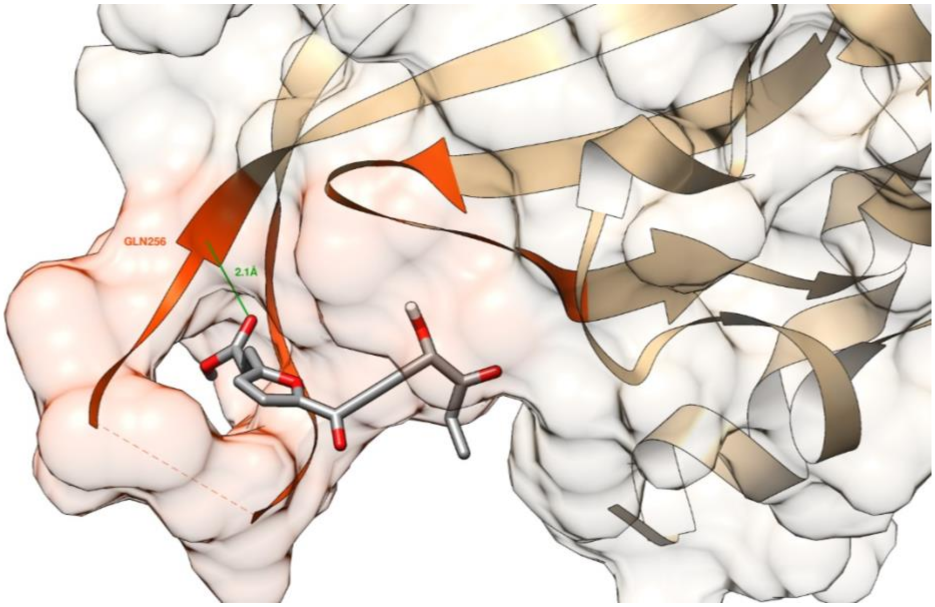

Figure 61. Binding mode analysis of methyl (E)-3-(5-(4-hydroxy-5-oxohept-2-ynoyl)furan-2-yl)acrylate (a metabolite derived from metabolism of wyerone epoxide) in the pocket 1 of AhR PAS-A domain.

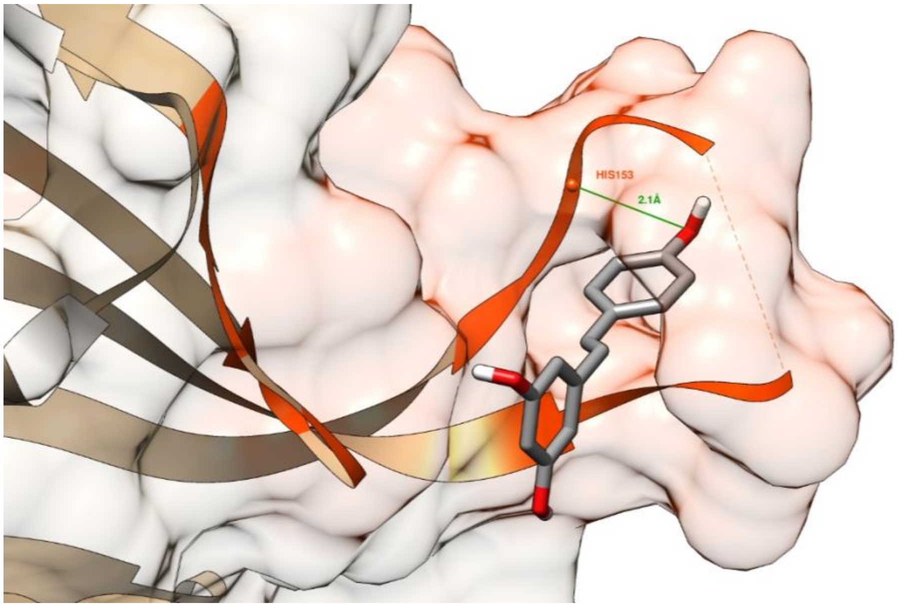

Figure 62. Binding mode analysis of resveratrol in the pocket 1 of AhR PAS-A domain.

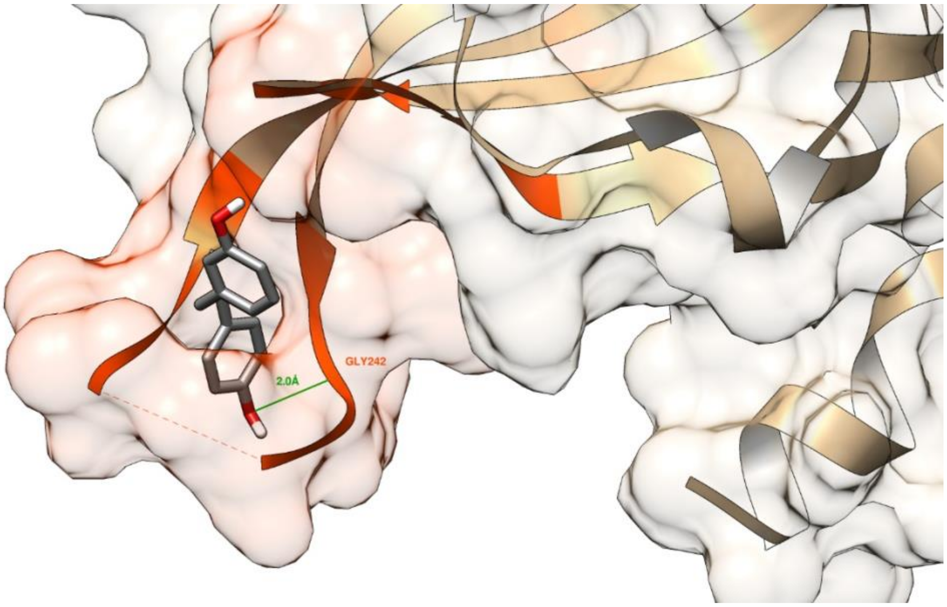

Figure 63. Binding mode analysis of lunarin (a metabolite derived from metabolism of resveratrol) in the pocket 1 of AhR PAS-A domain.

Reference

Pettersen, E. F., Goddard, T. D., Huang, C. C., Couch, G. S., Greenblatt, D. M., Meng, E. C., & Ferrin, T. E. (2004). UCSF Chimera—a visualization system for exploratory research and analysis. Journal of computational chemistry, *25*(13), 1605-1612.
